# Comparison of oxidation in uni-directionally and randomly oriented Cu films for low temperature Cu-to-Cu direct bonding

**DOI:** 10.1038/s41598-018-28812-0

**Published:** 2018-07-13

**Authors:** Chih-Han Tseng, K. N. Tu, Chih Chen

**Affiliations:** 1 Department of Materials Science and Engineering, National Chiao Tung University, Hsinchu, Taiwan 30010 Republic of China; 2 International College of Semiconductor Technology, National Chiao Tung University, Hsinchu, Taiwan 30010 Republic of China; 3 Department of Materials Science and Engineering, UCLA, Los Angeles, CA USA

## Abstract

Cu-to-Cu direct bonding has attracted attention because it has been implemented in CMOS image sensors. Prior to the bonding, the oxides on the Cu surface needs to be removed, yet the surface may oxidize right after cleaning. Thus, oxidation is an inherent issue in the application of Cu direct bonding. Our previous study reported that Cu direct bonding can be achieved below 250 °C by using (111)-oriented nanotwinned Cu because it has the fastest surface diffusivity. However, the oxidation behavior of the nanotwinned Cu is unclear. Here, we examined the oxidation behavior of highly (111) and (200) oriented, and randomly-oriented Cu films at temperatures ranging from 120 to 250 °C. Transmission electron microscopy was used to measure the oxide thickness. The results show that the oxidation rate of (111)-oriented nanotwinned Cu has the lowest oxidation rate among them. Together, it is unique to possess the combination of the fastest surface diffusivity and the lowest oxidation rate.

## Introduction

The microstructure of nanotwinned copper (nt-Cu) has attracted a large amount of research due to its unique mechanical, electrical, and chemical properties when compared with bulk Cu^1–5^. This is because Cu has been the most important interconnect conductor in advanced solid state electronics^6–11^. Moreover, Cu has a good wettability by molten solder, so it has been used widely as Under-Bump-Metallization (UBM) in microelectronic packaging technology. However, due to the need of low power and the reduction of Joule heating, Cu-to-Cu direct bonding (without solder) has been attempted and found that it can be achieved under a compressive stress of 100 psi. The bonding temperature can be lowered to 150–250 °C, which is below the solder joining temperature, and the bonding time is 10–60 min in low vacuum level^12,13^. The dominant parameter in the low temperature bonding is the fast surface diffusivity on Cu (111) surface. However, surface oxidation is expected to influence greatly the success in Cu-to-Cu direct bonding. The annealing of Cu films during bonding in ambient will produce an oxide layer, which may grow too fast or too thick and cause yield and reliability issues^14^.

On oxidation of metals, many kinetic theories exist for low-temperature oxidation^15–19^. The Wagner theory cannot fully explain the driving mechanism^20^. Other theories attempted to fix the mechanism of low temperature oxide growth. A majority of them was based on the Cabrera–Mott theory^16^. For example, Young presented that the much faster oxidation rate occurs on randomly oriented Cu, followed by (111) and (110) Cu at 70–178 °C^21^. Hu investigated the behavior of dynamics at 100–600 °C^22^. Zhou and Yang used the ultra-high-vacuum Transmission Electron Microscopy (UHV-TEM) to observe the oxide growth and they reported that the initial oxidation kinetics on (110) are faster than that on (100) at 350 °C^23,24^. Kusano found that the key factor affects the oxidation rate was the oxide thickness not the oxidation temperature, and presented the same tendency for all single crystals of Cu from 40–160 °C^25^.

Because the oxidation layer is thin, it is difficult to acquire accurate experimental data in thickness measurement. Therefore, we use TEM to observe the whole oxide layer, focus ion beam (FIB) to view the surface morphology, and X-ray photoelectron spectroscopy (XPS, VG Scientific Microlab 350) to analyze the Cu oxide. The roughness of the electrodeposited Cu film was analyzed with atomic force microscopy (AFM, diInnova Scanning Probe Microscope, Bruker). We found that different orientations of Cu surface such as (111), (100), and random orientation can have different oxidation behavior. It can have a dramatic effect on Cu-to-Cu direct bonding.

## Results and Discussion

**Figure 1:**
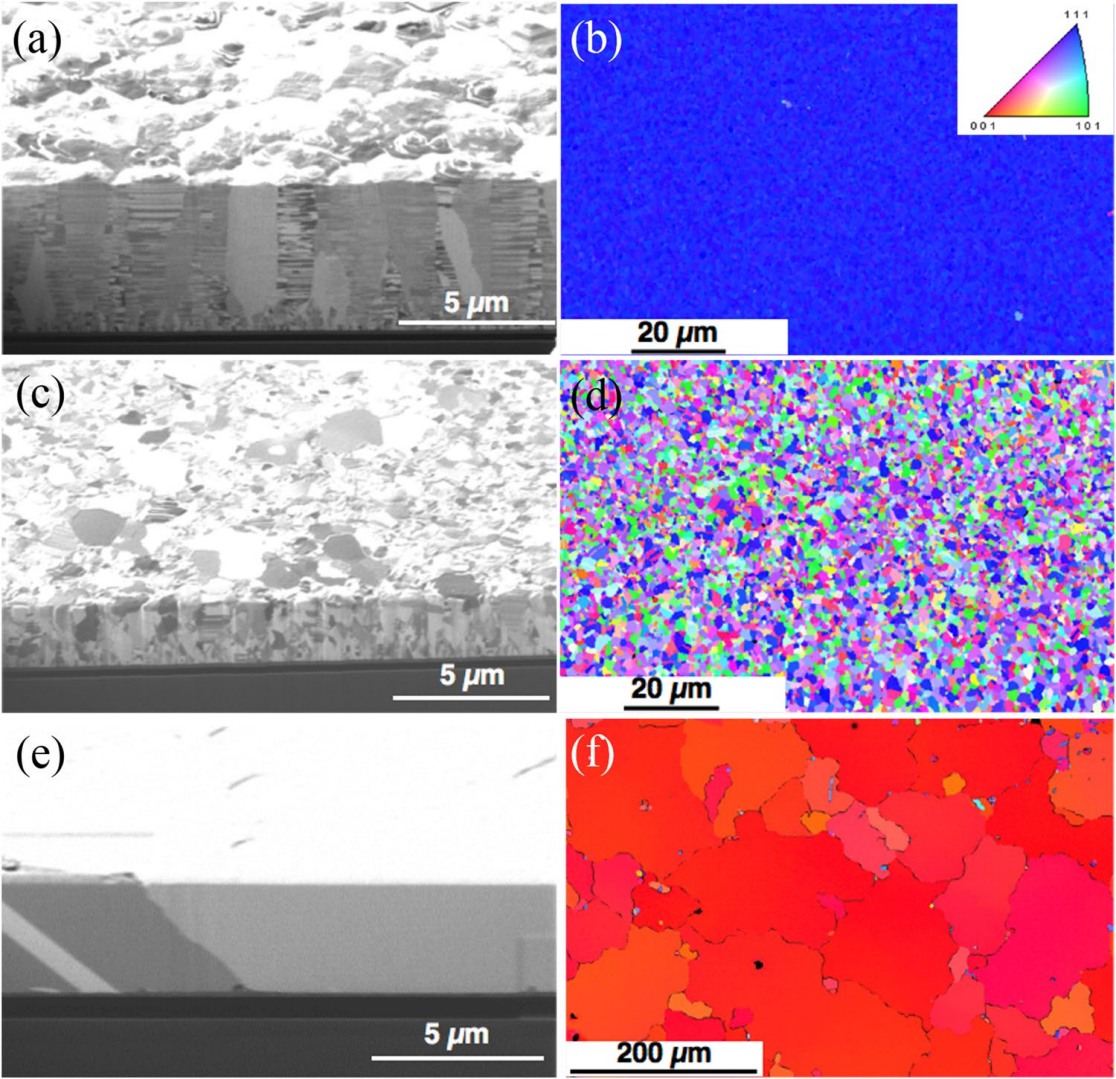
Preferred orientations of the three types of Cu films used in this study. (**a**) Cross-sectional FIB ion image for the highly (111)-oriented Cu film with nanotwins. (**b**) Plan-view EBSD OIM showing the (111)-oriented texture of the surface of the Cu film in (**a**). The inset shows the inverse pole figure. (**c**) Cross-sectional FIB ion image for the randomly-oriented Cu film. (**d**) Plan-view EBSD OIM for the texture of the Cu surface in (**c**). (**e**) Cross-sectional FIB ion image for the (200)-oriented Cu film. (**f**) Plan-view EBSD OIM showing the highly (200) preferred orientation for the Cu film in (**e**).

Before oxidation, the microstructure of sample was confirmed by EBSD and FIB. Figure 1a showed the cross-sectional FIB ion image of columnar grains in the electroplated nt-Cu films with (111) preferred orientation. We then used the EBSD to confirm the texture of grains on the surface. From Fig. 1b, we observed that the entire surface is blue, indicating the surface to have a 99.8% of (111) preferred orientation. In contrast, the microstructure of the random Cu film is quite different, in Fig. 1c. The corresponding plan-view EBSD orientation image map (OIM) were random, without any preferred orientation in Fig. 1d. The microstructure of (200) preferred Cu film is shown in Fig. 1e, where the Cu grains are very large, about 140 µm. The grain size and preferred orientation has been analyzed by EBSD as shown in Fig. 1f, where 98% of the surface was covered by (200) grains.

**Figure 2:**
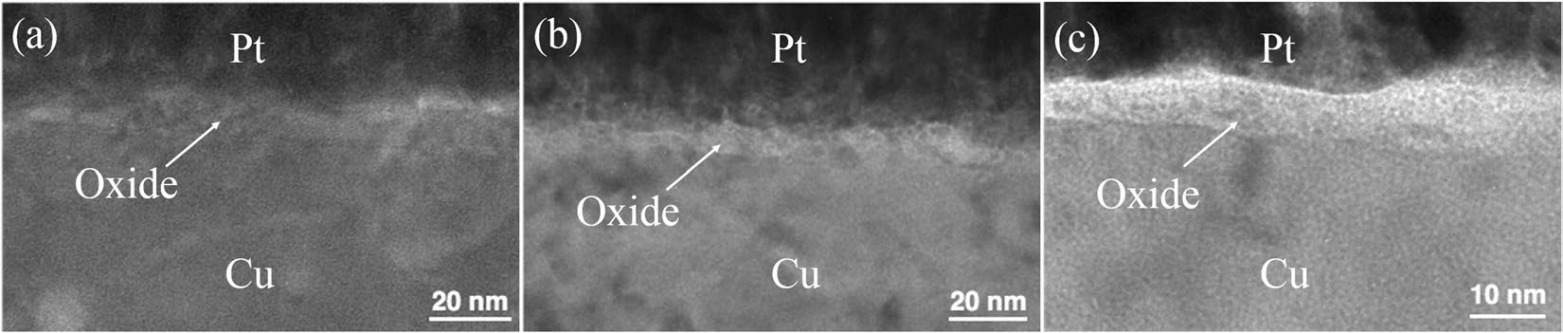
Cross-sectional TEM images showing the Cu oxide thickness after oxidation at 120 °C for 30 min. (**a**) Highly (111)-oriented nt-Cu film. (**b**) Randomly-oriented Cu film. (**c**) Highly (200)-oriented Cu film.

**Figure 3:**
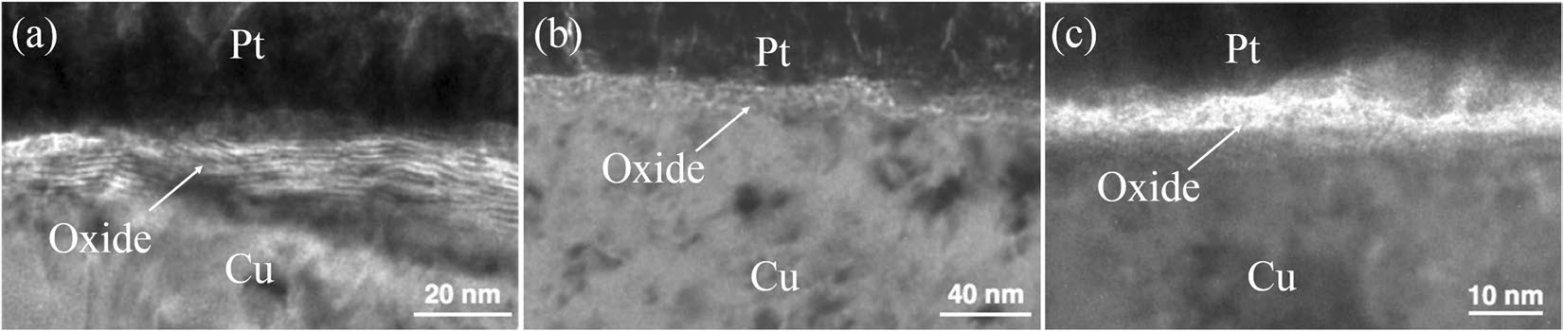
Cross-sectional TEM images showing the Cu oxide thickness after oxidation at 150 °C for 30 min. (**a**) Highly (111)-oriented nt-Cu film. (**b**) Randomly-oriented Cu film. (**c**) Highly (200)-oriented Cu film.

The TEM image of highly (111)-oriented nt-Cu film after oxidation at 120 °C for 30 min is shown in Fig. 2a. The bottom region is un-oxidized Cu film. The top region is covered by Pt and the middle region is Cu oxide. The thickness of the oxide layer was about 5.8 nm. For the randomly-oriented Cu, the thickness of oxide layer was 6.6 nm as shown in Fig. 2b. For the highly (200)-oriented Cu film, the oxide thickness was 6.5 nm as shown in Fig. 2c. When the oxidizing temperature was elevated to 150 °C for 30 minutes, the oxide thickness has changed slightly. The TEM images of this oxidation condition were shown in Fig. 3.

**Figure 4:**
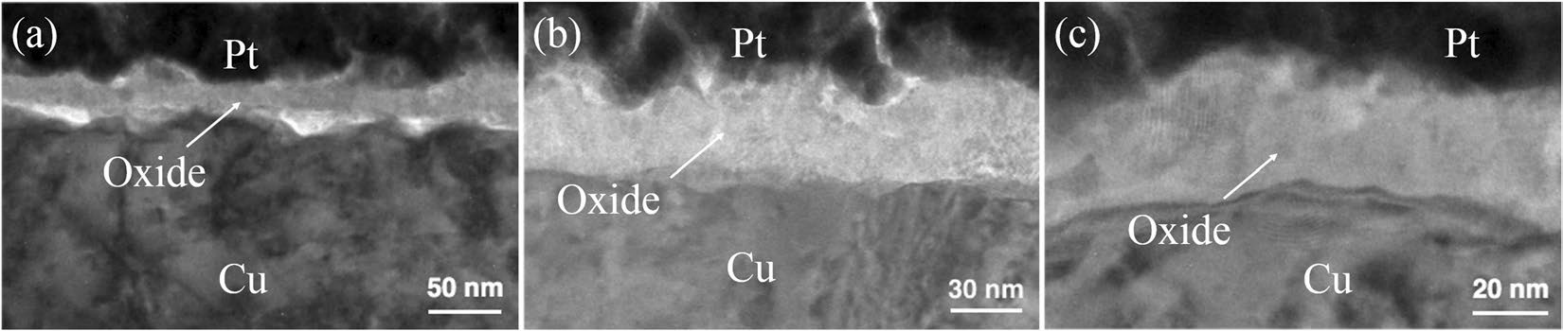
Cross-sectional TEM images showing the Cu oxide thickness after oxidation at 200 °C for 30 min. (**a**) Highly (111)-oriented nt-Cu film. (**b**) Randomly-oriented Cu film. (**c**) Highly (200)-oriented Cu film.

**Figure 5:**
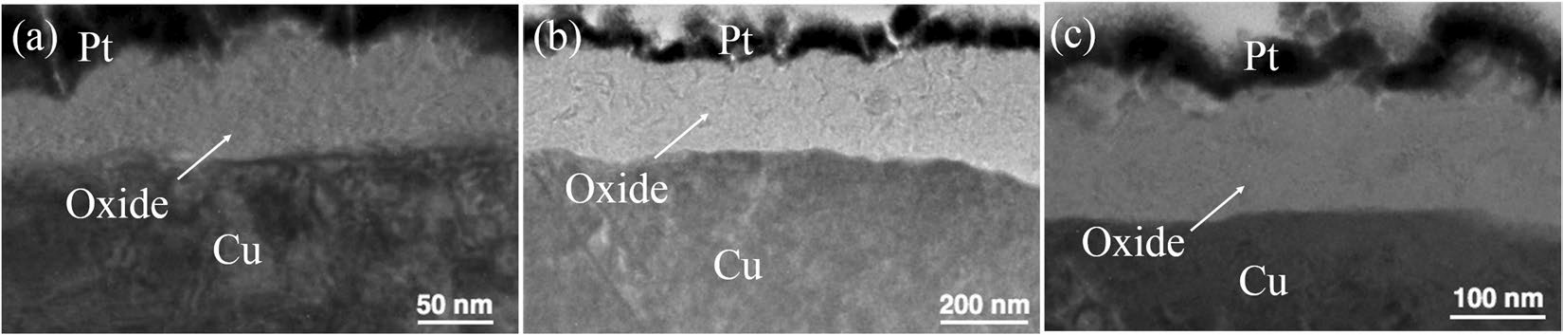
Cross-sectional TEM images showing the Cu oxide thickness after oxidation at 225 °C for 30 min. (**a**) Highly (111)-oriented nt-Cu film. (**b**) Randomly-oriented Cu film. (**c**) Highly (200)-oriented Cu film.

The oxide thicknesses measured at temperatures of 120, 150, 200, 225, 238, and 250 °C for 30 min are given in Table 1. When the oxidizing temperature was increased to 200 °C, the thickness increased greatly. The oxide thickness of highly (111)-oriented nt-Cu film is about 40 nm as shown in Fig. 4a. It was about 45 nm of highly (200)-oriented Cu film in Fig. 4c. But it was about 51.1 nm of the randomly-oriented Cu in Fig. 4b. When the oxidation temperature was further elevated to 225 °C for 30 minutes, Fig. 5a showed that the oxide thickness of highly (111)-oriented nt-Cu film did not change greatly and the oxide thickness was 68.5 nm. However, the oxide on the other preferred orientations was much thicker and more importantly their oxide growth mode was non-linear. The oxide thickness of highly (200)-oriented Cu film was about 150 nm as shown in Fig. 5c. The oxide thickness of randomly-oriented was about 240 nm in Fig. 5b. In comparison, the highly (111)-oriented nt-Cu film can effectively decrease the oxide thickness under high temperatures.

**Table 1:** Thickness of Cu oxide layer for different textures subjected to various annealing temperatures for 30 min.

	120 °C	150 °C	200 °C	225 °C	238 °C	250 °C
(111)	5.8 ± 0.3	9.3 ± 0.7	40.0 ± 1.8	68.5 ± 2.4	120.3 ± 2.4	245.1 ± 3.3
(100)	6.5 ± 0.8	9.9 ± 0.7	45.0 ± 1.5	147.6 ± 2.4	169.3 ± 3.8	456.2 ± 8.7
Random	6.6 ± 0.3	11.9 ± 0.4	51.1 ± 3.7	238.9 ± 8.0	501.6 ± 3.2	563.3 ± 9.2

The unit for thickness is nanometer.

**Figure 6:**
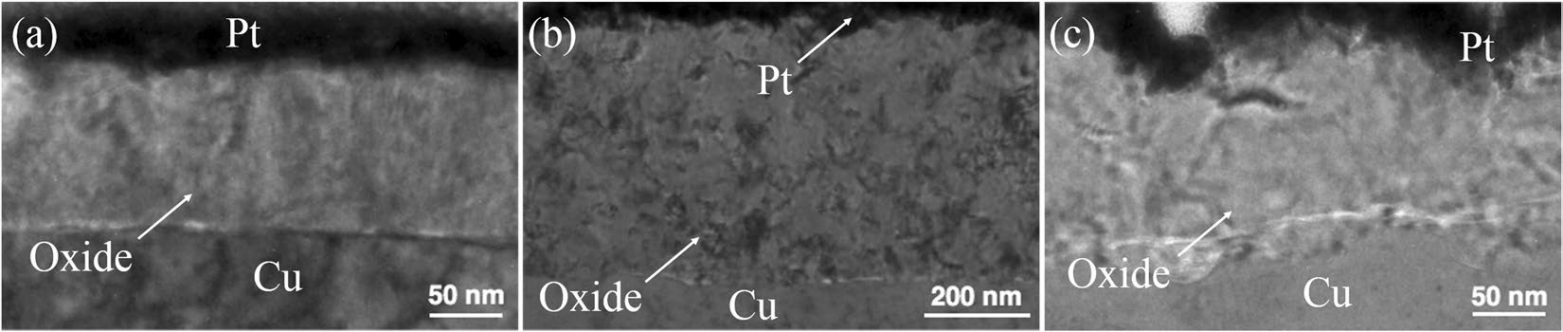
Cross-sectional TEM images showing the Cu oxide thickness after oxidation at 238 °C for 30 min. (**a**) Highly (111)-oriented nt-Cu film. (**b**) Randomly-oriented Cu film. (**c**) Highly (200)-oriented Cu film.

To determine the turning point of the oxidation rate, the oxidation temperature was kept at 238 °C. The oxide thickness difference among the three different orientations became even greater, as shown in Table 1. Figure 6a showed the change of oxide thickness of highly (111)-oriented nt-Cu film.

**Figure 7:**
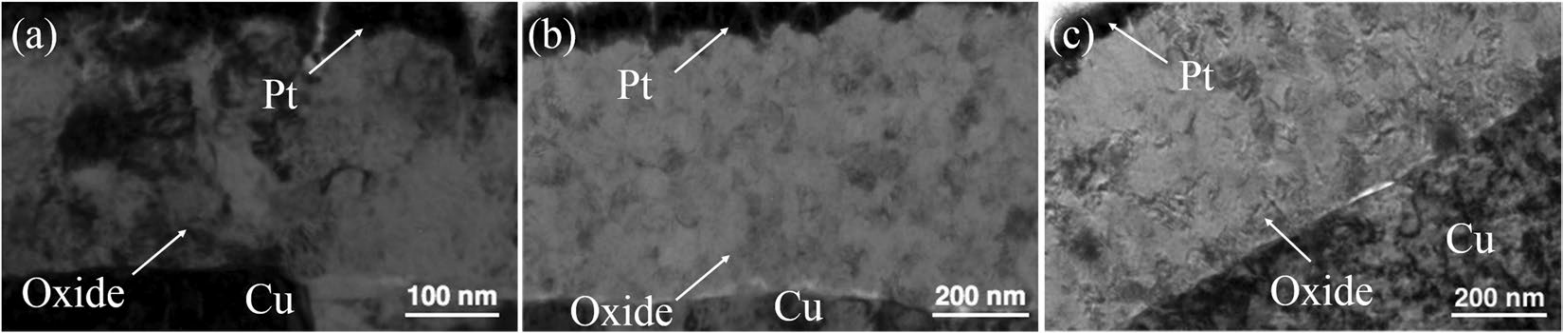
Cross-sectional TEM images showing the Cu oxide thickness after oxidation at 250 °C for 30 min. (**a**) Highly (111)-oriented nt-Cu film. (**b**) Randomly-oriented Cu film. (**c**) Highly (200)-oriented Cu film.

Finally, it is interesting to know whether the oxide thickness of the highly (111)-oriented nt-Cu film could be stable under high temperature. The image in Fig. 7 showed samples oxidized at 250 °C for 30 minutes. The oxide thickness of highly (111)-oriented nt-Cu film was below 250 nm in Fig. 7a. Then, the oxide thickness of highly (200)-oriented Cu film and the randomly-oriented was about 430 nm and 560 nm as shown in Fig. 7c,b, respectively.

**Figure 8:**
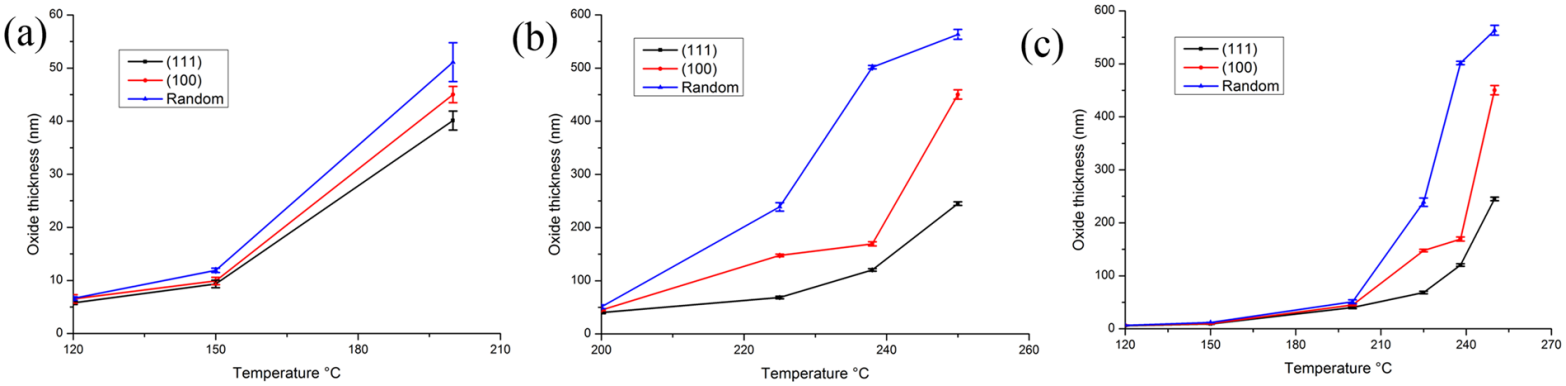
Plot of oxide thickness versus oxidation temperature for Cu films with different preferred orientation. (**a**) for temperature ranges from 120 °C to 200 °C (**b**) for temperature ranges from 200 °C to 250 °C (**c**) for all temperature ranges in this study.

We found that the critical oxidation temperature was 200 °C, where oxidation rate changes. Below 200 °C, the oxide growth mode is linear as shown in Fig. 8a. Above 200 °C, the mode becomes exponential as shown in Fig. 8b. On different oriented Cu films, the tendency of low temperature oxidation was the same. However, when the oxidation temperature was enhanced to above 200 °C, the tendency of different orientations is very different. This transition can be seen in Fig. 8c as well as in Table 1. Especially, the oxide thickness of highly (111)-oriented nt-Cu film is greatly reduced under high temperature.

**Figure 9:**
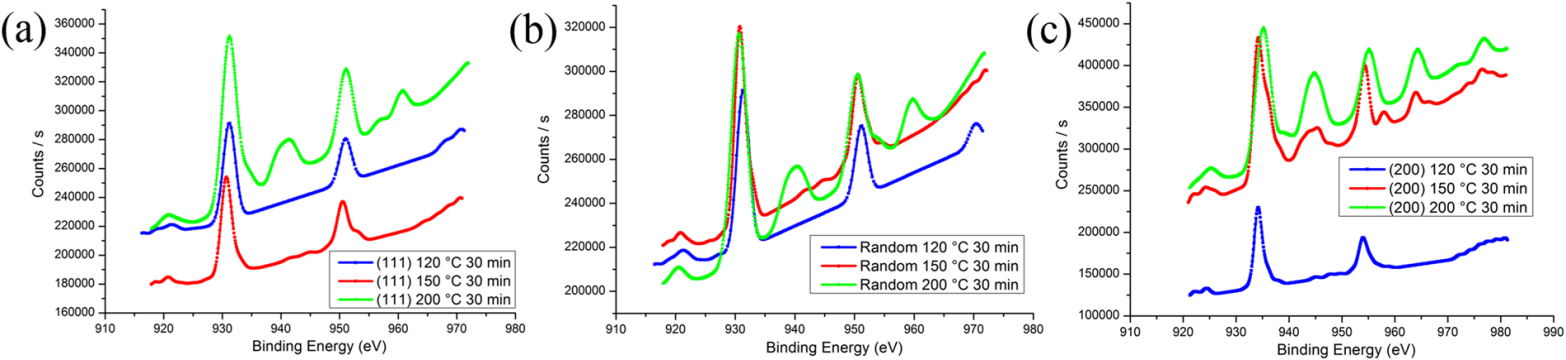
XPS surface analyses for the Cu films after oxidation tests. (**a**) Highly (111)-oriented nt-Cu film. (**b**) Randomly-oriented Cu film. (**c**) Highly (200)-oriented Cu film. The original data for the XPS were listed in the supplementary material.

The XPS was used to analyze whether the Cu oxide was CuO or Cu_2_O and the results are depicted in Fig. 9. First, we sputtered a thin layer of Pt to make a standard reference. Based on the binding energy database^26^, the binding energy of Cu 2p_1/2_ orbital was 952.5 eV and the other binding energy of Cu 2p_3/2_ orbital was 932.2 eV. When the Cu films were oxidized below 200 °C, the product was mainly Cu_2_O and a slight amount of CuO. But for the (200)-oriented Cu film, XPS results show obvious amounts of CuO. When the oxidation temperature was increased to 200 °C, both Cu_2_O and CuO will show an obvious amount for all the three samples.

**Figure 10:**
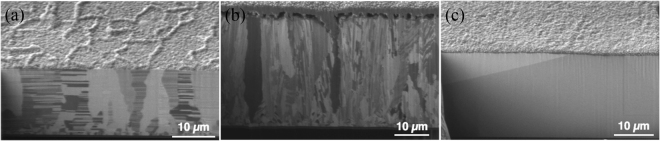
Cross-sectional FIB ion image of the electroplated Cu films with different preferred orientations after the oxidation at 200 °C for 120 min. (**a**) Highly (111)-oriented nt-Cu film. (**b**) Randomly-oriented Cu film. (**c**) Highly (200)-oriented Cu film.

**Figure 11:**
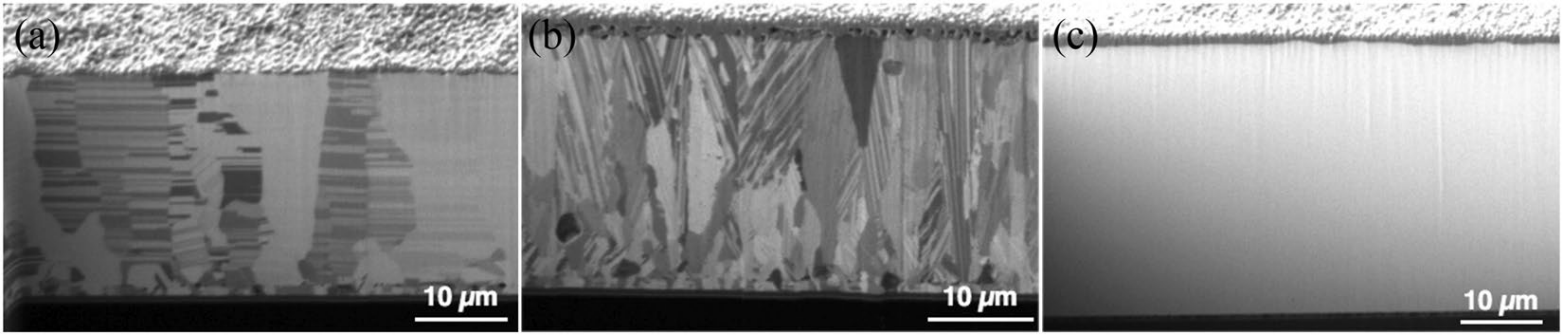
Cross-sectional FIB ion image images showing the oxidation thickness of the Cu films with different orientations after the oxidation at 200 °Cfor 240 min. (**a**) Highly (111)-oriented nt-Cu film. (**b**) Randomly-oriented Cu film. (**c**) Highly (200)-oriented Cu film.

The FIB result was shown in Figs 10 and 11, where we can analyze a larger area image than the TEM image. A new set of samples was oxidized at 200 °C for 120 minutes, and we found small voids appeared at the interface between the seed layer and electroplated Cu in Fig. 10a,b. However, only the highly (200)-oriented Cu film had no interfacial voids as shown in Fig. 10c. To verify it, the previous sample was oxidized at 200 °C for another 120 minutes. We used FIB to clean the region and the focused ion microscopy image was presented in Fig. 11, where large voids did appear at the interface between seed layer and electroplated copper. We expect that it is because Cu atoms can diffuse through the grain boundaries to the surface. We calculate the grain boundary diffusivity using the equation D = D_0_exp(−∆H/k_B_T). From previous studies^27,28^, the pre-factor of diffusivity of Cu in Cu grain boundary is D_0_ = 0.0232 cm^2^/s, and the activation enthalpy is ∆H = 84.75 kJ/mol. At 200 °C, the average diffusivity of Cu in Cu grain boundaries is 9.97 ∗ 10^−12^ cm^2^/s. For 120 minutes, the diffusion length was 2.76 μm on the basis of the equation ${\rm{X}}=\sqrt{{\rm{Dt}}}$. In the two-step annealing process of 120 minutes +120 minutes, the diffusion length wa 5.34 μm. From this calculation, we confirm that the grain boundary diffusion was reasonable in explaining the void formation.

On the oxide growth mode, a rather detailed discussion on linear and non-linear rates has been given, see Fig. 1 in ref.^29^, especially the rate dependence on oxide thickness on single crystal of (111) Cu. We will not repeat it here, instead, we emphasize that the (111) surface of Cu has the lowest oxidation rate as shown in Table 1.

## Conclusions

We used three different preferred orientations for oxide growth at different temperatures. The oxide was determined as Cu_2_O by XPS. The oxidation rate of the highly (111)-oriented nt-Cu film was especially low compared to the randomly-oriented Cu film and the highly (200)-oriented Cu film. When the oxidation temperature was below 200 °C, the oxidation rate was linear. When the oxidation temperature was above 200 °C, the mode becomes exponential. During oxidation, some small voids appear at the interface between the seed layer and Cu by grain boundary diffusion. Finally, we expect that the highly (111)-oriented nt-Cu film can facilitate low temperature direct Cu-to-Cu bonding because it possesses the fastest surface diffusivity and the lowest oxidation rate.

## Methods

We prepared three different samples. The first was the electroplated nt-Cu films with uni-directional (111) orientation, the second had (200) preferred orientation, and the third was electroplated Cu films with random orientation. To fabricate the (111)-oriented Cu film on a silicon wafer we sputtered a 100 nm thick Ti barrier layer and followed by a 200 nm thick Cu seed layer by Oerlikon ClusterLine 300. The Cu seed layer has a (111) preferred orientation. The silicon wafers were then cut into pieces of 1 × 3 cm^2^. Before electroplating, the pieces were cleaned with isopropyl alcohol and acetone in order to remove organic contaminations, and immersed into citric acid solution to remove surface oxides. The electroplating solution used was a high-purity CuSO_4_ solution plus additives. High stirring rate during the process is required for the fabrication of nt-Cu films with (111) preferred orientation. A stirring magnet was used for the stirring rate of 1200 rpm. The Cu films were deposited at room temperature. The direct current density was set at 80 mA cm^−2^. Another Cu sample with random orientation was fabricated by the same electrodeposition conditions but the electroplating bath was without additives. To fabricate the sample with (200) preferred orientation, we place the (111) preferred orientation copper films with nanotwin into the furnace and annealed it at 400 °C for one hour. Abnormal grain growth of (100) oriented grains occurred.

To prepare the samples for oxidation, the surface of the three kinds of as-electrodeposited Cu films mentioned in the above was smoothed by electro-polishing. The surface roughness, Ra, is 4.6 nm, 5.8 nm, and 3.2 nm for the (111) nt-Cu, random, and (200) Cu films, respectively. After cleaning the native surface oxides by immersing into citric acid solution, the samples were put on the hot plate at atmospheric pressure. The oxygen partial pressure was 101 kPa. The range of oxidation temperature was 120 °C to 250 °C and the oxidation time was 30 min. At the end of the oxidation, a thin layer of Pt film was sputtered on the Cu films to prevent further oxidation. Then, we used UHV-TEM to measure the oxide thickness. The microstructure of the electrodeposited Cu film was analyzed with a dual-beam focused ion beam (DB-FIB, FEI NOVA200). The UHV-TEM samples were prepared by dual-beam focused ion beam (DB-FIB, SEIKO SMI3050SE). We used Electron Backscatter Diffraction (EBSD, JEOL 7800 F field emission scanning electron microscope with an Oxford system) to analyze the grain orientation and grain size of the Cu films. We used the X-ray photoelectron spectroscopy (XPS, VG Scientific Microlab 350) to analyze the Cu oxide.

## Electronic supplementary material

Supplementary Dataset 1
