# Supplementary material for: Comparison of oxidation in uni-directionally and randomly oriented Cu films for low temperature Cu-to-Cu direct bonding
Source: Sci Rep. 2018 Jul 13;8:10671. doi: 10.1038/s41598-018-28812-0 (PMC6045573; doi:10.1038/s41598-018-28812-0)
Supplement: Supplementary file 1 — Supplementary Dataset 1 [file 41598_2018_28812_MOESM1_ESM.pdf]

Supplementary information

Ref.: SREP-18-02297

"Comparison of oxidation in uni-directionally and randomly oriented Cu films for low-temperature Cu-to-Cu direct bonding" by Chih-Han Tseng[1], K. N. Tu[2, 3] and Chih Chen[1]

XPS Raw data for Figure 9a: (111) nt-Cu.

XY	*	X Value	Y Value	Y Predict	Residual	Residual%	95% Confidence Limits		95% Prediction Limits		Weights
1		916.30000	2.16e+05	2.152e+05	761.57260	0.3526093	2.105e+05	2.199e+05	2.08e+05	2.224e+05	1
2		916.55000	2.163e+05	2.154e+05	896.13571	0.4143041	2.124e+05	2.184e+05	2.091e+05	2.217e+05	1
3		916.80000	2.164e+05	2.153e+05	1035.5056	0.4786208	2.124e+05	2.182e+05	2.091e+05	2.215e+05	1
4		917.05000	2.163e+05	2.151e+05	1104.9080	0.5109307	2.121e+05	2.182e+05	2.089e+05	2.214e+05	1
5		917.30000	2.159e+05	2.151e+05	770.15090	0.3567925	2.123e+05	2.179e+05	2.089e+05	2.213e+05	1
6		917.55000	2.159e+05	2.152e+05	669.19843	0.3099375	2.127e+05	2.178e+05	2.092e+05	2.213e+05	1
7		917.80000	2.158e+05	2.157e+05	120.94149	0.0560475	2.13e+05	2.183e+05	2.095e+05	2.218e+05	1
8		918.05000	2.174e+05	2.163e+05	1129.2447	0.5194198	2.134e+05	2.191e+05	2.101e+05	2.225e+05	1
9		918.30000	2.178e+05	2.17e+05	881.46645	0.4046412	2.142e+05	2.197e+05	2.108e+05	2.231e+05	1
10		918.55000	2.186e+05	2.176e+05	995.79954	0.4556017	2.15e+05	2.201e+05	2.115e+05	2.237e+05	1
11		918.80000	2.186e+05	2.18e+05	599.26787	0.2741089	2.154e+05	2.207e+05	2.119e+05	2.241e+05	1
12		919.05000	2.187e+05	2.183e+05	396.03409	0.1810938	2.155e+05	2.211e+05	2.121e+05	2.245e+05	1
13		919.30000	2.193e+05	2.184e+05	847.72848	0.3865928	2.157e+05	2.212e+05	2.123e+05	2.246e+05	1
14		919.55000	2.192e+05	2.185e+05	637.88185	0.2910244	2.159e+05	2.212e+05	2.125e+05	2.246e+05	1
15		919.80000	2.195e+05	2.187e+05	802.45372	0.3655193	2.161e+05	2.214e+05	2.126e+05	2.248e+05	1
16		920.05000	2.194e+05	2.191e+05	372.07970	0.1695642	2.163e+05	2.218e+05	2.129e+05	2.252e+05	1
17		920.30000	2.195e+05	2.195e+05	-1.245461	-0.000567	2.167e+05	2.223e+05	2.133e+05	2.257e+05	1
18		920.55000	2.21e+05	2.2e+05	972.08486	0.4398236	2.174e+05	2.227e+05	2.139e+05	2.262e+05	1
19		920.80000	2.212e+05	2.205e+05	692.42808	0.3129901	2.179e+05	2.232e+05	2.144e+05	2.267e+05	1
20		921.05000	2.214e+05	2.209e+05	496.42326	0.2242251	2.181e+05	2.237e+05	2.147e+05	2.271e+05	1
21		921.30000	2.217e+05	2.211e+05	660.56617	0.2979254	2.182e+05	2.239e+05	2.149e+05	2.273e+05	1
22		921.55000	2.216e+05	2.21e+05	578.27263	0.2609722	2.183e+05	2.237e+05	2.149e+05	2.271e+05	1
23		921.80000	2.211e+05	2.208e+05	373.74739	0.1690193	2.181e+05	2.234e+05	2.146e+05	2.269e+05	1
24		922.05000	2.204e+05	2.204e+05	94.037219	0.0426575	2.176e+05	2.231e+05	2.142e+05	2.265e+05	1
25		922.30000	2.195e+05	2.199e+05	-316.2640	-0.144052	2.17e+05	2.227e+05	2.137e+05	2.261e+05	1
26		922.55000	2.201e+05	2.193e+05	739.45628	0.3359867	2.167e+05	2.22e+05	2.132e+05	2.255e+05	1
27		922.80000	2.198e+05	2.188e+05	996.69690	0.4533614	2.162e+05	2.215e+05	2.127e+05	2.25e+05	1
28		923.05000	2.19e+05	2.184e+05	577.24558	0.2635849	2.157e+05	2.212e+05	2.123e+05	2.246e+05	1
29		923.30000	2.186e+05	2.181e+05	527.23507	0.2411618	2.153e+05	2.209e+05	2.119e+05	2.243e+05	1
30		923.55000	2.178e+05	2.179e+05	-65.42823	-0.030036	2.152e+05	2.206e+05	2.118e+05	2.24e+05	1

31	923.80000	2.174e+05	2.178e+05	-431.0197	-0.198266	2.153e+05	2.204e+05	2.117e+05	2.239e+05	1
32	924.05000	2.182e+05	2.179e+05	325.98413	0.1494017	2.152e+05	2.205e+05	2.117e+05	2.24e+05	1
33	924.30000	2.183e+05	2.18e+05	279.01863	0.1278354	2.152e+05	2.207e+05	2.118e+05	2.241e+05	1
34	924.55000	2.182e+05	2.181e+05	32.842382	0.0150535	2.155e+05	2.208e+05	2.12e+05	2.243e+05	1
35	924.80000	2.188e+05	2.183e+05	519.52724	0.2374341	2.158e+05	2.208e+05	2.122e+05	2.244e+05	1
36	925.05000	2.191e+05	2.184e+05	715.70579	0.3266094	2.158e+05	2.21e+05	2.123e+05	2.245e+05	1
37	925.30000	2.188e+05	2.185e+05	236.72859	0.1082183	2.158e+05	2.212e+05	2.124e+05	2.246e+05	1
38	925.55000	2.186e+05	2.186e+05	15.621883	0.0071460	2.159e+05	2.213e+05	2.125e+05	2.247e+05	1
39	925.80000	2.185e+05	2.187e+05	-200.2247	-0.091647	2.162e+05	2.212e+05	2.126e+05	2.247e+05	1
40	926.05000	2.189e+05	2.188e+05	152.58350	0.0696961	2.164e+05	2.212e+05	2.128e+05	2.248e+05	1
41	926.30000	2.185e+05	2.189e+05	-443.5592	-0.203040	2.164e+05	2.214e+05	2.128e+05	2.25e+05	1
42	926.55000	2.188e+05	2.191e+05	-243.8635	-0.111446	2.165e+05	2.217e+05	2.13e+05	2.252e+05	1
43	926.80000	2.191e+05	2.192e+05	-113.2640	-0.051687	2.167e+05	2.218e+05	2.132e+05	2.253e+05	1
44	927.05000	2.198e+05	2.195e+05	375.35800	0.1707430	2.171e+05	2.218e+05	2.135e+05	2.255e+05	1
45	927.30000	2.196e+05	2.197e+05	-60.69427	-0.027633	2.174e+05	2.22e+05	2.138e+05	2.257e+05	1
46	927.55000	2.202e+05	2.2e+05	170.01341	0.0772149	2.177e+05	2.224e+05	2.14e+05	2.26e+05	1
47	927.80000	2.209e+05	2.204e+05	430.11331	0.1947483	2.18e+05	2.228e+05	2.144e+05	2.264e+05	1
48	928.05000	2.218e+05	2.21e+05	753.77833	0.3398460	2.187e+05	2.234e+05	2.151e+05	2.27e+05	1
49	928.30000	2.23e+05	2.22e+05	947.32308	0.4248581	2.199e+05	2.241e+05	2.161e+05	2.279e+05	1
50	928.55000	2.239e+05	2.236e+05	327.05475	0.1460601	2.218e+05	2.254e+05	2.178e+05	2.294e+05	1
51	928.80000	2.251e+05	2.26e+05	-943.5640	-0.419198	2.245e+05	2.276e+05	2.203e+05	2.317e+05	1
52	929.05000	2.273e+05	2.297e+05	-2367.996	-1.041729	2.281e+05	2.312e+05	2.24e+05	2.354e+05	1
53	929.30000	2.311e+05	2.349e+05	-3744.679	-1.620330	2.331e+05	2.366e+05	2.291e+05	2.406e+05	1
54	929.55000	2.355e+05	2.417e+05	-6168.670	-2.618859	2.396e+05	2.438e+05	2.358e+05	2.476e+05	1
55	929.80000	2.441e+05	2.502e+05	-6104.627	-2.500861	2.479e+05	2.525e+05	2.442e+05	2.562e+05	1
56	930.05000	2.566e+05	2.599e+05	-3295.972	-1.284529	2.576e+05	2.622e+05	2.539e+05	2.659e+05	1
57	930.30000	2.704e+05	2.699e+05	417.59059	0.1544607	2.677e+05	2.722e+05	2.64e+05	2.759e+05	1
58	930.55000	2.846e+05	2.792e+05	5415.8732	1.9026964	2.77e+05	2.815e+05	2.733e+05	2.852e+05	1
59	930.80000	2.935e+05	2.865e+05	6951.5966	2.3687911	2.842e+05	2.888e+05	2.805e+05	2.925e+05	1
60	931.05000	2.957e+05	2.907e+05	4936.6659	1.6696879	2.883e+05	2.931e+05	2.847e+05	2.967e+05	1
61	931.30000	2.909e+05	2.912e+05	-366.2556	-0.125921	2.888e+05	2.936e+05	2.852e+05	2.972e+05	1
62	931.55000	2.844e+05	2.88e+05	-3557.323	-1.250680	2.857e+05	2.903e+05	2.82e+05	2.94e+05	1
63	931.80000	2.75e+05	2.816e+05	-6575.338	-2.390867	2.793e+05	2.838e+05	2.756e+05	2.875e+05	1
64	932.05000	2.679e+05	2.731e+05	-5152.861	-1.923154	2.708e+05	2.753e+05	2.671e+05	2.79e+05	1
65	932.30000	2.604e+05	2.637e+05	-3267.971	-1.254755	2.614e+05	2.66e+05	2.577e+05	2.697e+05	1
66	932.55000	2.536e+05	2.546e+05	-1026.125	-0.404639	2.523e+05	2.569e+05	2.487e+05	2.606e+05	1
67	932.80000	2.485e+05	2.466e+05	1844.9922	0.7424755	2.445e+05	2.488e+05	2.407e+05	2.525e+05	1
68	933.05000	2.439e+05	2.403e+05	3609.8927	1.4801800	2.384e+05	2.421e+05	2.345e+05	2.461e+05	1
69	933.30000	2.404e+05	2.356e+05	4774.5314	1.9863589	2.341e+05	2.371e+05	2.299e+05	2.413e+05	1

70	933.55000	2.369e+05	2.324e+05	4485.4612	1.8931908	2.313e+05	2.336e+05	2.268e+05	2.381e+05	1
71	933.80000	2.345e+05	2.305e+05	3948.1550	1.6839139	2.295e+05	2.315e+05	2.249e+05	2.361e+05	1
72	934.05000	2.329e+05	2.295e+05	3418.0044	1.4676098	2.286e+05	2.304e+05	2.239e+05	2.351e+05	1
73	934.30000	2.324e+05	2.29e+05	3410.9792	1.4674476	2.282e+05	2.299e+05	2.235e+05	2.346e+05	1
74	934.55000	2.311e+05	2.289e+05	2136.3881	0.9245031	2.281e+05	2.298e+05	2.234e+05	2.345e+05	1
75	934.80000	2.305e+05	2.291e+05	1419.4430	0.6158344	2.282e+05	2.3e+05	2.235e+05	2.347e+05	1
76	935.05000	2.294e+05	2.293e+05	97.187013	0.0423656	2.284e+05	2.302e+05	2.237e+05	2.349e+05	1
77	935.30000	2.293e+05	2.296e+05	-250.0986	-0.109051	2.287e+05	2.305e+05	2.24e+05	2.352e+05	1
78	935.55000	2.301e+05	2.299e+05	217.64980	0.0945814	2.29e+05	2.308e+05	2.243e+05	2.355e+05	1
79	935.80000	2.292e+05	2.302e+05	-982.4027	-0.428546	2.293e+05	2.311e+05	2.246e+05	2.358e+05	1
80	936.05000	2.298e+05	2.305e+05	-741.8352	-0.322807	2.297e+05	2.314e+05	2.25e+05	2.361e+05	1
81	936.30000	2.296e+05	2.309e+05	-1229.934	-0.535574	2.3e+05	2.318e+05	2.253e+05	2.365e+05	1
82	936.55000	2.295e+05	2.312e+05	-1734.629	-0.755922	2.303e+05	2.321e+05	2.256e+05	2.368e+05	1
83	936.80000	2.289e+05	2.315e+05	-2624.523	-1.146526	2.307e+05	2.324e+05	2.26e+05	2.371e+05	1
84	937.05000	2.292e+05	2.319e+05	-2622.480	-1.143979	2.31e+05	2.327e+05	2.263e+05	2.374e+05	1
85	937.30000	2.293e+05	2.322e+05	-2934.457	-1.279974	2.314e+05	2.33e+05	2.266e+05	2.378e+05	1
86	937.55000	2.301e+05	2.325e+05	-2442.438	-1.061560	2.317e+05	2.334e+05	2.27e+05	2.381e+05	1
87	937.80000	2.308e+05	2.329e+05	-2036.421	-0.882274	2.32e+05	2.337e+05	2.273e+05	2.384e+05	1
88	938.05000	2.305e+05	2.332e+05	-2654.404	-1.151455	2.324e+05	2.34e+05	2.276e+05	2.387e+05	1
89	938.30000	2.309e+05	2.335e+05	-2628.387	-1.138416	2.327e+05	2.343e+05	2.279e+05	2.391e+05	1
90	938.55000	2.318e+05	2.338e+05	-2031.370	-0.876320	2.33e+05	2.346e+05	2.283e+05	2.394e+05	1
91	938.80000	2.318e+05	2.342e+05	-2362.353	-1.019112	2.334e+05	2.349e+05	2.286e+05	2.397e+05	1
92	939.05000	2.32e+05	2.345e+05	-2510.336	-1.082107	2.337e+05	2.353e+05	2.289e+05	2.401e+05	1
93	939.30000	2.324e+05	2.348e+05	-2446.320	-1.052728	2.341e+05	2.356e+05	2.293e+05	2.404e+05	1
94	939.55000	2.33e+05	2.352e+05	-2169.303	-0.931091	2.344e+05	2.359e+05	2.296e+05	2.407e+05	1
95	939.80000	2.326e+05	2.355e+05	-2875.286	-1.236108	2.347e+05	2.362e+05	2.299e+05	2.41e+05	1
96	940.05000	2.33e+05	2.358e+05	-2794.269	-1.199164	2.351e+05	2.365e+05	2.303e+05	2.414e+05	1
97	940.30000	2.337e+05	2.361e+05	-2479.252	-1.061042	2.354e+05	2.369e+05	2.306e+05	2.417e+05	1
98	940.55000	2.342e+05	2.365e+05	-2222.235	-0.948668	2.358e+05	2.372e+05	2.309e+05	2.42e+05	1
99	940.80000	2.351e+05	2.368e+05	-1706.219	-0.725763	2.361e+05	2.375e+05	2.312e+05	2.424e+05	1
100	941.05000	2.35e+05	2.371e+05	-2143.202	-0.912059	2.364e+05	2.378e+05	2.316e+05	2.427e+05	1
101	941.30000	2.361e+05	2.375e+05	-1396.185	-0.591451	2.368e+05	2.381e+05	2.319e+05	2.43e+05	1
102	941.55000	2.365e+05	2.378e+05	-1260.168	-0.532782	2.371e+05	2.385e+05	2.322e+05	2.433e+05	1
103	941.80000	2.368e+05	2.381e+05	-1318.151	-0.556659	2.374e+05	2.388e+05	2.326e+05	2.437e+05	1
104	942.05000	2.369e+05	2.384e+05	-1497.135	-0.631844	2.378e+05	2.391e+05	2.329e+05	2.44e+05	1
105	942.30000	2.373e+05	2.388e+05	-1476.118	-0.622055	2.381e+05	2.394e+05	2.332e+05	2.443e+05	1
106	942.55000	2.381e+05	2.391e+05	-966.1009	-0.405693	2.385e+05	2.398e+05	2.336e+05	2.447e+05	1
107	942.80000	2.386e+05	2.394e+05	-858.0840	-0.359674	2.388e+05	2.401e+05	2.339e+05	2.45e+05	1
108	943.05000	2.385e+05	2.398e+05	-1250.067	-0.524115	2.391e+05	2.404e+05	2.342e+05	2.453e+05	1

109	943.30000	2.387e+05	2.401e+05	-1432.050	-0.600045	2.395e+05	2.407e+05	2.345e+05	2.456e+05	1
110	943.55000	2.384e+05	2.404e+05	-2021.034	-0.847760	2.398e+05	2.41e+05	2.349e+05	2.46e+05	1
111	943.80000	2.395e+05	2.407e+05	-1231.017	-0.513960	2.401e+05	2.414e+05	2.352e+05	2.463e+05	1
112	944.05000	2.4e+05	2.411e+05	-1084.000	-0.451682	2.405e+05	2.417e+05	2.355e+05	2.466e+05	1
113	944.30000	2.4e+05	2.414e+05	-1393.983	-0.580800	2.408e+05	2.42e+05	2.359e+05	2.469e+05	1
114	944.55000	2.401e+05	2.417e+05	-1627.966	-0.678020	2.411e+05	2.423e+05	2.362e+05	2.473e+05	1
115	944.80000	2.406e+05	2.421e+05	-1491.950	-0.620170	2.415e+05	2.427e+05	2.365e+05	2.476e+05	1
116	945.05000	2.403e+05	2.424e+05	-2096.935	-0.872650	2.418e+05	2.43e+05	2.369e+05	2.479e+05	1
117	945.30000	2.408e+05	2.427e+05	-1937.925	-0.804843	2.421e+05	2.433e+05	2.372e+05	2.483e+05	1
118	945.55000	2.413e+05	2.43e+05	-1750.932	-0.725628	2.425e+05	2.436e+05	2.375e+05	2.486e+05	1
119	945.80000	2.426e+05	2.434e+05	-741.9959	-0.305805	2.428e+05	2.44e+05	2.378e+05	2.489e+05	1
120	946.05000	2.425e+05	2.437e+05	-1228.231	-0.506529	2.431e+05	2.443e+05	2.382e+05	2.492e+05	1
121	946.30000	2.419e+05	2.44e+05	-2138.960	-0.884237	2.435e+05	2.446e+05	2.385e+05	2.496e+05	1
122	946.55000	2.424e+05	2.444e+05	-2012.019	-0.830188	2.438e+05	2.449e+05	2.388e+05	2.499e+05	1
123	946.80000	2.427e+05	2.447e+05	-2010.449	-0.828392	2.442e+05	2.453e+05	2.392e+05	2.502e+05	1
124	947.05000	2.436e+05	2.45e+05	-1486.880	-0.610480	2.445e+05	2.456e+05	2.395e+05	2.506e+05	1
125	947.30000	2.436e+05	2.454e+05	-1776.108	-0.729019	2.449e+05	2.459e+05	2.399e+05	2.509e+05	1
126	947.55000	2.439e+05	2.458e+05	-1949.367	-0.799399	2.453e+05	2.463e+05	2.403e+05	2.513e+05	1
127	947.80000	2.447e+05	2.463e+05	-1525.577	-0.623328	2.458e+05	2.468e+05	2.407e+05	2.518e+05	1
128	948.05000	2.456e+05	2.469e+05	-1276.060	-0.519577	2.464e+05	2.474e+05	2.413e+05	2.524e+05	1
129	948.30000	2.464e+05	2.477e+05	-1337.656	-0.542983	2.471e+05	2.483e+05	2.422e+05	2.532e+05	1
130	948.55000	2.476e+05	2.489e+05	-1210.191	-0.488690	2.482e+05	2.495e+05	2.433e+05	2.544e+05	1
131	948.80000	2.486e+05	2.505e+05	-1869.502	-0.751927	2.496e+05	2.514e+05	2.449e+05	2.561e+05	1
132	949.05000	2.514e+05	2.528e+05	-1360.398	-0.541086	2.516e+05	2.54e+05	2.471e+05	2.584e+05	1
133	949.30000	2.543e+05	2.558e+05	-1488.521	-0.585301	2.543e+05	2.573e+05	2.501e+05	2.615e+05	1
134	949.55000	2.578e+05	2.596e+05	-1827.625	-0.709052	2.578e+05	2.614e+05	2.538e+05	2.654e+05	1
135	949.80000	2.621e+05	2.64e+05	-1835.361	-0.700140	2.619e+05	2.66e+05	2.581e+05	2.699e+05	1
136	950.05000	2.679e+05	2.687e+05	-775.7650	-0.289575	2.665e+05	2.709e+05	2.627e+05	2.746e+05	1
137	950.30000	2.724e+05	2.732e+05	-851.6709	-0.312711	2.71e+05	2.754e+05	2.673e+05	2.791e+05	1
138	950.55000	2.762e+05	2.77e+05	-777.1180	-0.281327	2.748e+05	2.792e+05	2.711e+05	2.829e+05	1
139	950.80000	2.785e+05	2.796e+05	-1112.001	-0.399335	2.774e+05	2.817e+05	2.737e+05	2.855e+05	1
140	951.05000	2.775e+05	2.805e+05	-3079.345	-1.109846	2.784e+05	2.827e+05	2.746e+05	2.864e+05	1
141	951.30000	2.756e+05	2.798e+05	-4141.644	-1.502517	2.776e+05	2.819e+05	2.739e+05	2.857e+05	1
142	951.55000	2.732e+05	2.775e+05	-4328.655	-1.584531	2.753e+05	2.797e+05	2.716e+05	2.834e+05	1
143	951.80000	2.701e+05	2.741e+05	-4003.849	-1.482287	2.719e+05	2.763e+05	2.682e+05	2.801e+05	1
144	952.05000	2.672e+05	2.701e+05	-2914.638	-1.090665	2.68e+05	2.723e+05	2.642e+05	2.761e+05	1
145	952.30000	2.641e+05	2.662e+05	-2038.889	-0.771979	2.642e+05	2.682e+05	2.603e+05	2.72e+05	1
146	952.55000	2.626e+05	2.626e+05	1.7550642	0.0006685	2.608e+05	2.643e+05	2.568e+05	2.683e+05	1
147	952.80000	2.609e+05	2.596e+05	1315.9927	0.5043239	2.582e+05	2.611e+05	2.539e+05	2.653e+05	1

148	953.05000	2.594e+05	2.575e+05	1922.6002	0.7412206	2.563e+05	2.586e+05	2.518e+05	2.631e+05	1
149	953.30000	2.583e+05	2.56e+05	2329.1225	0.9015551	2.551e+05	2.569e+05	2.504e+05	2.616e+05	1
150	953.55000	2.573e+05	2.552e+05	2085.0357	0.8104937	2.545e+05	2.559e+05	2.496e+05	2.607e+05	1
151	953.80000	2.566e+05	2.548e+05	1784.2654	0.6954682	2.542e+05	2.554e+05	2.492e+05	2.603e+05	1
152	954.05000	2.555e+05	2.547e+05	804.65427	0.3149529	2.541e+05	2.552e+05	2.491e+05	2.602e+05	1
153	954.30000	2.544e+05	2.548e+05	-329.4272	-0.129466	2.542e+05	2.553e+05	2.492e+05	2.603e+05	1
154	954.55000	2.546e+05	2.55e+05	-397.7019	-0.156210	2.544e+05	2.556e+05	2.495e+05	2.605e+05	1
155	954.80000	2.549e+05	2.553e+05	-373.7849	-0.146645	2.547e+05	2.558e+05	2.497e+05	2.608e+05	1
156	955.05000	2.547e+05	2.556e+05	-910.5978	-0.357577	2.55e+05	2.562e+05	2.5e+05	2.611e+05	1
157	955.30000	2.544e+05	2.559e+05	-1484.920	-0.583691	2.553e+05	2.565e+05	2.503e+05	2.614e+05	1
158	955.55000	2.548e+05	2.562e+05	-1448.654	-0.568628	2.556e+05	2.568e+05	2.507e+05	2.618e+05	1
159	955.80000	2.544e+05	2.565e+05	-2147.043	-0.843990	2.559e+05	2.571e+05	2.51e+05	2.621e+05	1
160	956.05000	2.545e+05	2.569e+05	-2397.463	-0.942140	2.563e+05	2.575e+05	2.513e+05	2.624e+05	1
161	956.30000	2.544e+05	2.572e+05	-2795.258	-1.098761	2.566e+05	2.578e+05	2.517e+05	2.627e+05	1
162	956.55000	2.54e+05	2.575e+05	-3544.183	-1.395452	2.569e+05	2.582e+05	2.52e+05	2.631e+05	1
163	956.80000	2.55e+05	2.579e+05	-2839.148	-1.113326	2.572e+05	2.585e+05	2.523e+05	2.634e+05	1
164	957.05000	2.553e+05	2.582e+05	-2890.127	-1.132082	2.575e+05	2.588e+05	2.526e+05	2.637e+05	1
165	957.30000	2.558e+05	2.585e+05	-2684.109	-1.049185	2.579e+05	2.592e+05	2.53e+05	2.641e+05	1
166	957.55000	2.557e+05	2.588e+05	-3101.092	-1.212595	2.582e+05	2.595e+05	2.533e+05	2.644e+05	1
167	957.80000	2.556e+05	2.592e+05	-3586.075	-1.403090	2.585e+05	2.598e+05	2.536e+05	2.647e+05	1
168	958.05000	2.557e+05	2.595e+05	-3778.058	-1.477414	2.588e+05	2.602e+05	2.539e+05	2.651e+05	1
169	958.30000	2.553e+05	2.598e+05	-4533.041	-1.775609	2.591e+05	2.605e+05	2.543e+05	2.654e+05	1
170	958.55000	2.555e+05	2.602e+05	-4646.024	-1.818326	2.595e+05	2.608e+05	2.546e+05	2.657e+05	1
171	958.80000	2.564e+05	2.605e+05	-4106.007	-1.601532	2.598e+05	2.612e+05	2.549e+05	2.66e+05	1
172	959.05000	2.57e+05	2.608e+05	-3769.990	-1.466666	2.601e+05	2.615e+05	2.553e+05	2.664e+05	1
173	959.30000	2.569e+05	2.611e+05	-4247.974	-1.653577	2.604e+05	2.619e+05	2.556e+05	2.667e+05	1
174	959.55000	2.568e+05	2.615e+05	-4647.957	-1.809776	2.608e+05	2.622e+05	2.559e+05	2.67e+05	1
175	959.80000	2.575e+05	2.618e+05	-4318.940	-1.677369	2.611e+05	2.625e+05	2.562e+05	2.674e+05	1
176	960.05000	2.567e+05	2.621e+05	-5403.923	-2.104930	2.614e+05	2.629e+05	2.566e+05	2.677e+05	1
177	960.30000	2.575e+05	2.625e+05	-4972.906	-1.931323	2.617e+05	2.632e+05	2.569e+05	2.68e+05	1
178	960.55000	2.581e+05	2.628e+05	-4719.890	-1.828925	2.62e+05	2.635e+05	2.572e+05	2.684e+05	1
179	960.80000	2.585e+05	2.631e+05	-4614.873	-1.785230	2.624e+05	2.639e+05	2.576e+05	2.687e+05	1
180	961.05000	2.589e+05	2.634e+05	-4585.856	-1.771551	2.627e+05	2.642e+05	2.579e+05	2.69e+05	1
181	961.30000	2.591e+05	2.638e+05	-4703.839	-1.815649	2.63e+05	2.646e+05	2.582e+05	2.693e+05	1
182	961.55000	2.589e+05	2.641e+05	-5177.822	-1.999723	2.633e+05	2.649e+05	2.585e+05	2.697e+05	1
183	961.80000	2.597e+05	2.644e+05	-4708.805	-1.812997	2.636e+05	2.652e+05	2.589e+05	2.7e+05	1
184	962.05000	2.607e+05	2.648e+05	-4052.789	-1.554520	2.639e+05	2.656e+05	2.592e+05	2.703e+05	1
185	962.30000	2.611e+05	2.651e+05	-3995.773	-1.530385	2.643e+05	2.659e+05	2.595e+05	2.707e+05	1
186	962.55000	2.616e+05	2.654e+05	-3796.759	-1.451227	2.646e+05	2.663e+05	2.598e+05	2.71e+05	1

187	962.80000	2.619e+05	2.657e+05	-3882.751	-1.482719	2.649e+05	2.666e+05	2.602e+05	2.713e+05	1
188	963.05000	2.625e+05	2.661e+05	-3560.765	-1.356389	2.652e+05	2.669e+05	2.605e+05	2.717e+05	1
189	963.30000	2.637e+05	2.664e+05	-2711.840	-1.028396	2.655e+05	2.673e+05	2.608e+05	2.72e+05	1
190	963.55000	2.64e+05	2.667e+05	-2756.088	-1.044048	2.659e+05	2.676e+05	2.612e+05	2.723e+05	1
191	963.80000	2.646e+05	2.671e+05	-2515.784	-0.950964	2.662e+05	2.68e+05	2.615e+05	2.726e+05	1
192	964.05000	2.646e+05	2.674e+05	-2794.578	-1.056140	2.665e+05	2.683e+05	2.618e+05	2.73e+05	1
193	964.30000	2.65e+05	2.677e+05	-2770.892	-1.045777	2.668e+05	2.686e+05	2.621e+05	2.733e+05	1
194	964.55000	2.655e+05	2.681e+05	-2521.614	-0.949589	2.672e+05	2.69e+05	2.625e+05	2.737e+05	1
195	964.80000	2.662e+05	2.684e+05	-2230.191	-0.837822	2.675e+05	2.693e+05	2.628e+05	2.74e+05	1
196	965.05000	2.671e+05	2.688e+05	-1691.090	-0.633135	2.679e+05	2.697e+05	2.632e+05	2.744e+05	1
197	965.30000	2.675e+05	2.692e+05	-1707.391	-0.638308	2.683e+05	2.701e+05	2.636e+05	2.748e+05	1
198	965.55000	2.677e+05	2.697e+05	-1957.872	-0.731371	2.687e+05	2.706e+05	2.641e+05	2.752e+05	1
199	965.80000	2.681e+05	2.702e+05	-2145.555	-0.800404	2.692e+05	2.712e+05	2.646e+05	2.758e+05	1
200	966.05000	2.685e+05	2.709e+05	-2367.510	-0.881751	2.697e+05	2.721e+05	2.652e+05	2.765e+05	1
201	966.30000	2.692e+05	2.717e+05	-2472.064	-0.918287	2.702e+05	2.731e+05	2.66e+05	2.774e+05	1
202	966.55000	2.703e+05	2.726e+05	-2381.844	-0.881319	2.709e+05	2.744e+05	2.668e+05	2.784e+05	1
203	966.80000	2.709e+05	2.738e+05	-2817.893	-1.040066	2.717e+05	2.758e+05	2.679e+05	2.796e+05	1
204	967.05000	2.713e+05	2.75e+05	-3654.062	-1.346812	2.727e+05	2.772e+05	2.69e+05	2.809e+05	1
205	967.30000	2.719e+05	2.762e+05	-4308.519	-1.584597	2.739e+05	2.785e+05	2.702e+05	2.822e+05	1
206	967.55000	2.736e+05	2.774e+05	-3766.768	-1.376652	2.751e+05	2.796e+05	2.714e+05	2.833e+05	1
207	967.80000	2.745e+05	2.784e+05	-3859.656	-1.405842	2.762e+05	2.806e+05	2.725e+05	2.843e+05	1
208	968.05000	2.753e+05	2.792e+05	-3949.767	-1.434943	2.77e+05	2.814e+05	2.733e+05	2.851e+05	1
209	968.30000	2.764e+05	2.798e+05	-3362.539	-1.216438	2.774e+05	2.822e+05	2.738e+05	2.858e+05	1
210	968.55000	2.767e+05	2.802e+05	-3555.725	-1.285256	2.777e+05	2.827e+05	2.742e+05	2.863e+05	1
211	968.80000	2.769e+05	2.806e+05	-3653.532	-1.319265	2.782e+05	2.83e+05	2.746e+05	2.866e+05	1
212	969.05000	2.778e+05	2.811e+05	-3251.971	-1.170569	2.788e+05	2.833e+05	2.751e+05	2.87e+05	1
213	969.30000	2.786e+05	2.817e+05	-3169.038	-1.137605	2.796e+05	2.839e+05	2.758e+05	2.877e+05	1
214	969.55000	2.809e+05	2.827e+05	-1807.111	-0.643420	2.804e+05	2.85e+05	2.767e+05	2.886e+05	1
215	969.80000	2.804e+05	2.838e+05	-3370.813	-1.202029	2.813e+05	2.863e+05	2.777e+05	2.899e+05	1
216	970.05000	2.807e+05	2.85e+05	-4304.834	-1.533650	2.824e+05	2.876e+05	2.789e+05	2.911e+05	1
217	970.30000	2.814e+05	2.861e+05	-4672.996	-1.660630	2.836e+05	2.886e+05	2.8e+05	2.921e+05	1
218	970.55000	2.825e+05	2.868e+05	-4355.440	-1.541907	2.845e+05	2.892e+05	2.808e+05	2.928e+05	1
219	970.80000	2.83e+05	2.871e+05	-4115.084	-1.454099	2.846e+05	2.896e+05	2.811e+05	2.932e+05	1
220	971.05000	2.835e+05	2.869e+05	-3422.964	-1.207578	2.838e+05	2.899e+05	2.806e+05	2.932e+05	1
221	971.30000	2.835e+05	2.862e+05	-2625.572	-0.925974	2.826e+05	2.898e+05	2.796e+05	2.928e+05	1

XPS Raw data for Figure 9b: random Cu

XY	*	X Value	Y Value	Y Predict	Residual	Residual%	95% Confidence Limits		95% Prediction Limits		Weights
1		916.50000	2.124e+05	2.122e+05	128.43573	0.0604796	2.086e+05	2.159e+05	2.067e+05	2.178e+05	1
2		916.75000	2.121e+05	2.125e+05	-395.3989	-0.186419	2.102e+05	2.148e+05	2.077e+05	2.173e+05	1
3		917.00000	2.127e+05	2.125e+05	245.84775	0.1155642	2.102e+05	2.148e+05	2.077e+05	2.173e+05	1
4		917.25000	2.125e+05	2.124e+05	134.29700	0.0631920	2.1e+05	2.148e+05	2.075e+05	2.172e+05	1
5		917.50000	2.123e+05	2.124e+05	-36.56719	-0.017223	2.102e+05	2.145e+05	2.076e+05	2.171e+05	1
6		917.75000	2.125e+05	2.125e+05	56.314663	0.0264953	2.104e+05	2.145e+05	2.078e+05	2.172e+05	1
7		918.00000	2.129e+05	2.128e+05	101.31832	0.0475836	2.106e+05	2.15e+05	2.081e+05	2.176e+05	1
8		918.25000	2.126e+05	2.133e+05	-714.6766	-0.336173	2.111e+05	2.155e+05	2.085e+05	2.181e+05	1
9		918.50000	2.139e+05	2.138e+05	93.774009	0.0438336	2.118e+05	2.159e+05	2.091e+05	2.185e+05	1
10		918.75000	2.148e+05	2.143e+05	423.61176	0.1972535	2.123e+05	2.164e+05	2.096e+05	2.19e+05	1
11		919.00000	2.151e+05	2.147e+05	332.57711	0.1546338	2.126e+05	2.169e+05	2.1e+05	2.195e+05	1
12		919.25000	2.151e+05	2.151e+05	-28.20348	-0.013115	2.129e+05	2.173e+05	2.103e+05	2.198e+05	1
13		919.50000	2.151e+05	2.154e+05	-321.9468	-0.149680	2.133e+05	2.175e+05	2.107e+05	2.201e+05	1
14		919.75000	2.156e+05	2.158e+05	-156.9179	-0.072768	2.138e+05	2.178e+05	2.111e+05	2.205e+05	1
15		920.00000	2.16e+05	2.163e+05	-312.2130	-0.144562	2.141e+05	2.184e+05	2.115e+05	2.21e+05	1
16		920.25000	2.172e+05	2.169e+05	371.30792	0.1709285	2.147e+05	2.19e+05	2.121e+05	2.216e+05	1
17		920.50000	2.175e+05	2.175e+05	72.924475	0.0335229	2.154e+05	2.196e+05	2.128e+05	2.222e+05	1
18		920.75000	2.182e+05	2.18e+05	228.33806	0.1046285	2.159e+05	2.201e+05	2.133e+05	2.227e+05	1
19		921.00000	2.183e+05	2.184e+05	-88.17189	-0.040387	2.162e+05	2.206e+05	2.137e+05	2.232e+05	1
20		921.25000	2.188e+05	2.186e+05	174.86855	0.0799322	2.164e+05	2.208e+05	2.138e+05	2.233e+05	1
21		921.50000	2.179e+05	2.186e+05	-660.1098	-0.302954	2.165e+05	2.206e+05	2.138e+05	2.233e+05	1
22		921.75000	2.184e+05	2.183e+05	121.76215	0.0557504	2.162e+05	2.204e+05	2.136e+05	2.23e+05	1
23		922.00000	2.179e+05	2.178e+05	101.31267	0.0464874	2.157e+05	2.2e+05	2.131e+05	2.226e+05	1
24		922.25000	2.174e+05	2.173e+05	153.94814	0.0708094	2.151e+05	2.194e+05	2.125e+05	2.22e+05	1
25		922.50000	2.166e+05	2.166e+05	-7.341484	-0.003389	2.146e+05	2.187e+05	2.119e+05	2.213e+05	1
26		922.75000	2.163e+05	2.16e+05	328.10571	0.1516831	2.139e+05	2.18e+05	2.113e+05	2.207e+05	1
27		923.00000	2.152e+05	2.154e+05	-176.3973	-0.081956	2.133e+05	2.175e+05	2.107e+05	2.201e+05	1
28		923.25000	2.144e+05	2.15e+05	-568.6415	-0.265246	2.128e+05	2.171e+05	2.102e+05	2.197e+05	1
29		923.50000	2.147e+05	2.146e+05	62.953132	0.0293224	2.126e+05	2.166e+05	2.1e+05	2.193e+05	1
30		923.75000	2.149e+05	2.144e+05	436.53999	0.2031571	2.125e+05	2.164e+05	2.098e+05	2.191e+05	1
31		924.00000	2.145e+05	2.144e+05	173.83390	0.0810301	2.123e+05	2.164e+05	2.097e+05	2.19e+05	1
32		924.25000	2.142e+05	2.143e+05	-95.60457	-0.044626	2.122e+05	2.164e+05	2.096e+05	2.19e+05	1
33		924.50000	2.142e+05	2.143e+05	-161.9845	-0.075636	2.123e+05	2.163e+05	2.097e+05	2.19e+05	1
34		924.75000	2.137e+05	2.143e+05	-592.3782	-0.277174	2.124e+05	2.162e+05	2.097e+05	2.189e+05	1
35		925.00000	2.143e+05	2.143e+05	43.671569	0.0203744	2.124e+05	2.162e+05	2.097e+05	2.189e+05	1
36		925.25000	2.151e+05	2.143e+05	775.82984	0.3607018	2.123e+05	2.163e+05	2.096e+05	2.19e+05	1
37		925.50000	2.144e+05	2.144e+05	47.542485	0.0221713	2.124e+05	2.164e+05	2.097e+05	2.19e+05	1

38	925.75000	2.148e+05	2.146e+05	222.81532	0.1037446	2.127e+05	2.164e+05	2.1e+05	2.191e+05	1
39	926.00000	2.149e+05	2.148e+05	112.29557	0.0522468	2.131e+05	2.165e+05	2.103e+05	2.194e+05	1
40	926.25000	2.145e+05	2.152e+05	-661.2566	-0.308245	2.134e+05	2.169e+05	2.106e+05	2.198e+05	1
41	926.50000	2.152e+05	2.156e+05	-435.5241	-0.202411	2.137e+05	2.175e+05	2.11e+05	2.202e+05	1
42	926.75000	2.158e+05	2.16e+05	-222.6819	-0.103187	2.141e+05	2.179e+05	2.114e+05	2.207e+05	1
43	927.00000	2.158e+05	2.164e+05	-582.9975	-0.270131	2.146e+05	2.182e+05	2.118e+05	2.21e+05	1
44	927.25000	2.166e+05	2.167e+05	-138.9411	-0.064158	2.15e+05	2.184e+05	2.121e+05	2.213e+05	1
45	927.50000	2.178e+05	2.169e+05	887.68496	0.4075576	2.152e+05	2.186e+05	2.124e+05	2.215e+05	1
46	927.75000	2.172e+05	2.171e+05	78.149399	0.0359834	2.154e+05	2.188e+05	2.125e+05	2.217e+05	1
47	928.00000	2.182e+05	2.174e+05	879.99815	0.4032323	2.155e+05	2.192e+05	2.128e+05	2.22e+05	1
48	928.25000	2.187e+05	2.178e+05	839.28754	0.3838059	2.161e+05	2.196e+05	2.133e+05	2.224e+05	1
49	928.50000	2.197e+05	2.188e+05	946.74305	0.4308881	2.171e+05	2.204e+05	2.143e+05	2.233e+05	1
50	928.75000	2.208e+05	2.205e+05	333.42963	0.1510112	2.191e+05	2.219e+05	2.16e+05	2.249e+05	1
51	929.00000	2.225e+05	2.233e+05	-815.5463	-0.366618	2.22e+05	2.245e+05	2.189e+05	2.277e+05	1
52	929.25000	2.258e+05	2.275e+05	-1701.542	-0.753425	2.263e+05	2.288e+05	2.231e+05	2.32e+05	1
53	929.50000	2.299e+05	2.336e+05	-3719.534	-1.618146	2.321e+05	2.351e+05	2.291e+05	2.381e+05	1
54	929.75000	2.365e+05	2.415e+05	-5002.783	-2.115467	2.398e+05	2.432e+05	2.37e+05	2.46e+05	1
55	930.00000	2.451e+05	2.51e+05	-5923.909	-2.416778	2.493e+05	2.528e+05	2.465e+05	2.556e+05	1
56	930.25000	2.591e+05	2.616e+05	-2510.811	-0.969036	2.598e+05	2.634e+05	2.57e+05	2.662e+05	1
57	930.50000	2.735e+05	2.722e+05	1273.6622	0.4657445	2.705e+05	2.739e+05	2.676e+05	2.768e+05	1
58	930.75000	2.872e+05	2.815e+05	5685.9617	1.9799227	2.798e+05	2.832e+05	2.769e+05	2.861e+05	1
59	931.00000	2.952e+05	2.882e+05	7018.4296	2.3772269	2.864e+05	2.9e+05	2.836e+05	2.928e+05	1
60	931.25000	2.961e+05	2.913e+05	4777.2258	1.6132409	2.895e+05	2.932e+05	2.867e+05	2.96e+05	1
61	931.50000	2.904e+05	2.904e+05	-57.26044	-0.019720	2.886e+05	2.923e+05	2.858e+05	2.95e+05	1
62	931.75000	2.818e+05	2.856e+05	-3864.609	-1.371528	2.839e+05	2.874e+05	2.811e+05	2.902e+05	1
63	932.00000	2.723e+05	2.778e+05	-5552.634	-2.039408	2.761e+05	2.795e+05	2.733e+05	2.824e+05	1
64	932.25000	2.626e+05	2.682e+05	-5569.375	-2.120754	2.665e+05	2.699e+05	2.636e+05	2.727e+05	1
65	932.50000	2.553e+05	2.58e+05	-2776.952	-1.087853	2.563e+05	2.598e+05	2.535e+05	2.626e+05	1
66	932.75000	2.482e+05	2.486e+05	-338.1782	-0.136246	2.468e+05	2.503e+05	2.44e+05	2.531e+05	1
67	933.00000	2.435e+05	2.405e+05	2979.5790	1.2238877	2.389e+05	2.42e+05	2.36e+05	2.45e+05	1
68	933.25000	2.39e+05	2.342e+05	4803.4721	2.0100144	2.329e+05	2.355e+05	2.298e+05	2.386e+05	1
69	933.50000	2.352e+05	2.297e+05	5587.9392	2.3753902	2.286e+05	2.307e+05	2.253e+05	2.34e+05	1
70	933.75000	2.318e+05	2.267e+05	5070.5262	2.1879293	2.258e+05	2.275e+05	2.224e+05	2.31e+05	1
71	934.00000	2.297e+05	2.249e+05	4835.5268	2.1048189	2.242e+05	2.256e+05	2.206e+05	2.292e+05	1
72	934.25000	2.289e+05	2.24e+05	4887.8388	2.1357891	2.233e+05	2.246e+05	2.197e+05	2.282e+05	1
73	934.50000	2.27e+05	2.236e+05	3438.0270	1.5144293	2.229e+05	2.242e+05	2.193e+05	2.279e+05	1
74	934.75000	2.268e+05	2.235e+05	3277.1872	1.4449679	2.229e+05	2.242e+05	2.193e+05	2.278e+05	1
75	935.00000	2.264e+05	2.236e+05	2740.8500	1.2106763	2.23e+05	2.243e+05	2.194e+05	2.279e+05	1
76	935.25000	2.262e+05	2.239e+05	2366.1434	1.0458693	2.232e+05	2.246e+05	2.196e+05	2.281e+05	1

77	935.50000	2.251e+05	2.241e+05	1003.1388	0.4455583	2.235e+05	2.248e+05	2.199e+05	2.284e+05	1
78	935.75000	2.243e+05	2.244e+05	-88.89657	-0.039626	2.238e+05	2.251e+05	2.202e+05	2.287e+05	1
79	936.00000	2.252e+05	2.247e+05	457.11700	0.2029980	2.241e+05	2.254e+05	2.205e+05	2.29e+05	1
80	936.25000	2.247e+05	2.25e+05	-308.4430	-0.137257	2.244e+05	2.257e+05	2.208e+05	2.293e+05	1
81	936.50000	2.255e+05	2.253e+05	150.65742	0.0668160	2.247e+05	2.26e+05	2.211e+05	2.296e+05	1
82	936.75000	2.257e+05	2.256e+05	113.28617	0.0501828	2.25e+05	2.263e+05	2.214e+05	2.299e+05	1
83	937.00000	2.249e+05	2.259e+05	-1050.241	-0.467008	2.253e+05	2.266e+05	2.217e+05	2.302e+05	1
84	937.25000	2.246e+05	2.262e+05	-1681.817	-0.748942	2.256e+05	2.269e+05	2.22e+05	2.305e+05	1
85	937.50000	2.246e+05	2.265e+05	-1905.408	-0.848209	2.259e+05	2.272e+05	2.223e+05	2.308e+05	1
86	937.75000	2.254e+05	2.268e+05	-1414.002	-0.627235	2.262e+05	2.275e+05	2.226e+05	2.311e+05	1
87	938.00000	2.253e+05	2.272e+05	-1898.597	-0.842873	2.265e+05	2.278e+05	2.229e+05	2.314e+05	1
88	938.25000	2.254e+05	2.275e+05	-2015.193	-0.893893	2.268e+05	2.281e+05	2.232e+05	2.317e+05	1
89	938.50000	2.256e+05	2.278e+05	-2132.788	-0.945276	2.272e+05	2.284e+05	2.235e+05	2.32e+05	1
90	938.75000	2.263e+05	2.281e+05	-1780.384	-0.786799	2.275e+05	2.287e+05	2.238e+05	2.323e+05	1
91	939.00000	2.268e+05	2.284e+05	-1581.979	-0.697571	2.278e+05	2.29e+05	2.241e+05	2.326e+05	1
92	939.25000	2.271e+05	2.287e+05	-1538.575	-0.677395	2.281e+05	2.293e+05	2.244e+05	2.329e+05	1
93	939.50000	2.274e+05	2.29e+05	-1601.171	-0.704207	2.284e+05	2.295e+05	2.247e+05	2.332e+05	1
94	939.75000	2.274e+05	2.293e+05	-1833.766	-0.806253	2.287e+05	2.298e+05	2.25e+05	2.335e+05	1
95	940.00000	2.277e+05	2.296e+05	-1857.362	-0.815623	2.29e+05	2.301e+05	2.253e+05	2.338e+05	1
96	940.25000	2.277e+05	2.299e+05	-2168.957	-0.952488	2.293e+05	2.304e+05	2.256e+05	2.341e+05	1
97	940.50000	2.29e+05	2.302e+05	-1232.553	-0.538339	2.296e+05	2.307e+05	2.259e+05	2.344e+05	1
98	940.75000	2.295e+05	2.305e+05	-972.1486	-0.423559	2.299e+05	2.31e+05	2.262e+05	2.347e+05	1
99	941.00000	2.301e+05	2.308e+05	-706.7442	-0.307163	2.303e+05	2.313e+05	2.265e+05	2.35e+05	1
100	941.25000	2.31e+05	2.311e+05	-128.3397	-0.055566	2.306e+05	2.316e+05	2.268e+05	2.354e+05	1
101	941.50000	2.314e+05	2.314e+05	-9.935337	-0.004294	2.309e+05	2.319e+05	2.272e+05	2.357e+05	1
102	941.75000	2.318e+05	2.317e+05	90.469072	0.0390296	2.312e+05	2.322e+05	2.275e+05	2.36e+05	1
103	942.00000	2.316e+05	2.32e+05	-430.1265	-0.185736	2.315e+05	2.325e+05	2.278e+05	2.363e+05	1
104	942.25000	2.328e+05	2.323e+05	477.27789	0.2050251	2.318e+05	2.328e+05	2.281e+05	2.366e+05	1
105	942.50000	2.321e+05	2.326e+05	-529.3177	-0.228069	2.321e+05	2.331e+05	2.284e+05	2.369e+05	1
106	942.75000	2.321e+05	2.329e+05	-842.9133	-0.363204	2.324e+05	2.334e+05	2.287e+05	2.372e+05	1
107	943.00000	2.328e+05	2.332e+05	-440.5089	-0.189236	2.327e+05	2.337e+05	2.29e+05	2.375e+05	1
108	943.25000	2.333e+05	2.335e+05	-189.1045	-0.081043	2.33e+05	2.34e+05	2.293e+05	2.378e+05	1
109	943.50000	2.337e+05	2.338e+05	-127.7001	-0.054642	2.333e+05	2.343e+05	2.296e+05	2.381e+05	1
110	943.75000	2.334e+05	2.341e+05	-696.2957	-0.298279	2.337e+05	2.346e+05	2.299e+05	2.384e+05	1
111	944.00000	2.336e+05	2.344e+05	-824.8913	-0.353102	2.34e+05	2.349e+05	2.302e+05	2.387e+05	1
112	944.25000	2.346e+05	2.347e+05	-119.4869	-0.050927	2.343e+05	2.352e+05	2.305e+05	2.39e+05	1
113	944.50000	2.353e+05	2.35e+05	275.91751	0.1172515	2.346e+05	2.355e+05	2.308e+05	2.393e+05	1
114	944.75000	2.357e+05	2.353e+05	312.32171	0.1325301	2.349e+05	2.358e+05	2.311e+05	2.396e+05	1
115	945.00000	2.361e+05	2.357e+05	403.72529	0.1710295	2.352e+05	2.361e+05	2.314e+05	2.399e+05	1

116	945.25000	2.365e+05	2.36e+05	535.12648	0.2262777	2.355e+05	2.364e+05	2.317e+05	2.402e+05	1
117	945.50000	2.364e+05	2.363e+05	137.51915	0.0581730	2.358e+05	2.367e+05	2.32e+05	2.405e+05	1
118	945.75000	2.367e+05	2.366e+05	104.88316	0.0443166	2.361e+05	2.37e+05	2.323e+05	2.408e+05	1
119	946.00000	2.368e+05	2.369e+05	-105.8435	-0.044705	2.364e+05	2.373e+05	2.326e+05	2.411e+05	1
120	946.25000	2.373e+05	2.372e+05	165.15986	0.0695890	2.367e+05	2.376e+05	2.329e+05	2.414e+05	1
121	946.50000	2.381e+05	2.375e+05	608.40808	0.2555435	2.37e+05	2.379e+05	2.332e+05	2.417e+05	1
122	946.75000	2.383e+05	2.378e+05	499.67171	0.2096976	2.374e+05	2.382e+05	2.335e+05	2.42e+05	1
123	947.00000	2.384e+05	2.381e+05	331.03962	0.1388443	2.377e+05	2.385e+05	2.339e+05	2.423e+05	1
124	947.25000	2.381e+05	2.384e+05	-311.9182	-0.131000	2.38e+05	2.388e+05	2.342e+05	2.427e+05	1
125	947.50000	2.391e+05	2.388e+05	291.58184	0.1219722	2.384e+05	2.392e+05	2.345e+05	2.43e+05	1
126	947.75000	2.405e+05	2.392e+05	1383.3385	0.5750851	2.388e+05	2.396e+05	2.349e+05	2.434e+05	1
127	948.00000	2.406e+05	2.397e+05	902.96753	0.3753669	2.393e+05	2.4e+05	2.354e+05	2.439e+05	1
128	948.25000	2.417e+05	2.403e+05	1410.7153	0.5836131	2.399e+05	2.407e+05	2.361e+05	2.445e+05	1
129	948.50000	2.419e+05	2.412e+05	696.11334	0.2877274	2.408e+05	2.417e+05	2.37e+05	2.455e+05	1
130	948.75000	2.434e+05	2.426e+05	791.42630	0.3251960	2.42e+05	2.431e+05	2.383e+05	2.468e+05	1
131	949.00000	2.45e+05	2.445e+05	521.15176	0.2127098	2.437e+05	2.452e+05	2.402e+05	2.488e+05	1
132	949.25000	2.473e+05	2.471e+05	162.61075	0.0657624	2.461e+05	2.481e+05	2.428e+05	2.514e+05	1
133	949.50000	2.503e+05	2.505e+05	-201.4848	-0.080488	2.493e+05	2.518e+05	2.461e+05	2.549e+05	1
134	949.75000	2.544e+05	2.547e+05	-280.4946	-0.110240	2.533e+05	2.562e+05	2.503e+05	2.592e+05	1
135	950.00000	2.597e+05	2.595e+05	249.94982	0.0962378	2.579e+05	2.611e+05	2.55e+05	2.64e+05	1
136	950.25000	2.651e+05	2.644e+05	720.11592	0.2716209	2.627e+05	2.661e+05	2.599e+05	2.689e+05	1
137	950.50000	2.701e+05	2.69e+05	1119.4878	0.4144931	2.673e+05	2.707e+05	2.644e+05	2.735e+05	1
138	950.75000	2.741e+05	2.726e+05	1490.1246	0.5436804	2.709e+05	2.743e+05	2.681e+05	2.771e+05	1
139	951.00000	2.753e+05	2.748e+05	565.74423	0.2054801	2.731e+05	2.764e+05	2.702e+05	2.793e+05	1
140	951.25000	2.74e+05	2.752e+05	-1152.594	-0.420618	2.736e+05	2.768e+05	2.707e+05	2.797e+05	1
141	951.50000	2.721e+05	2.738e+05	-1713.481	-0.629720	2.722e+05	2.755e+05	2.693e+05	2.783e+05	1
142	951.75000	2.686e+05	2.71e+05	-2339.162	-0.870833	2.693e+05	2.727e+05	2.664e+05	2.755e+05	1
143	952.00000	2.652e+05	2.671e+05	-1879.692	-0.708799	2.654e+05	2.688e+05	2.625e+05	2.716e+05	1
144	952.25000	2.621e+05	2.628e+05	-670.0231	-0.255639	2.611e+05	2.644e+05	2.582e+05	2.673e+05	1
145	952.50000	2.592e+05	2.586e+05	579.93871	0.2237789	2.571e+05	2.601e+05	2.541e+05	2.63e+05	1
146	952.75000	2.574e+05	2.549e+05	2442.9148	0.9492393	2.536e+05	2.562e+05	2.505e+05	2.593e+05	1
147	953.00000	2.556e+05	2.52e+05	3610.0170	1.4123255	2.51e+05	2.53e+05	2.477e+05	2.563e+05	1
148	953.25000	2.54e+05	2.499e+05	4066.2093	1.6011598	2.491e+05	2.507e+05	2.456e+05	2.542e+05	1
149	953.50000	2.524e+05	2.485e+05	3930.3120	1.5569415	2.479e+05	2.491e+05	2.442e+05	2.528e+05	1
150	953.75000	2.513e+05	2.477e+05	3611.4801	1.4369646	2.472e+05	2.482e+05	2.435e+05	2.52e+05	1
151	954.00000	2.504e+05	2.474e+05	3063.8556	1.2235063	2.469e+05	2.478e+05	2.431e+05	2.516e+05	1
152	954.25000	2.501e+05	2.473e+05	2817.6321	1.1266337	2.469e+05	2.477e+05	2.43e+05	2.515e+05	1
153	954.50000	2.504e+05	2.474e+05	2998.5570	1.1976311	2.47e+05	2.478e+05	2.431e+05	2.516e+05	1
154	954.75000	2.493e+05	2.476e+05	1705.2344	0.6840584	2.472e+05	2.48e+05	2.433e+05	2.518e+05	1

155	955.00000	2.494e+05	2.478e+05	1566.9478	0.6282895	2.474e+05	2.482e+05	2.436e+05	2.521e+05	1
156	955.25000	2.49e+05	2.481e+05	849.81552	0.3413407	2.477e+05	2.485e+05	2.439e+05	2.524e+05	1
157	955.50000	2.49e+05	2.484e+05	584.19438	0.2346228	2.48e+05	2.488e+05	2.442e+05	2.526e+05	1
158	955.75000	2.49e+05	2.487e+05	302.13189	0.1213327	2.483e+05	2.491e+05	2.445e+05	2.529e+05	1
159	956.00000	2.492e+05	2.49e+05	144.84640	0.0581348	2.486e+05	2.494e+05	2.448e+05	2.533e+05	1
160	956.25000	2.495e+05	2.493e+05	224.70852	0.0900495	2.489e+05	2.497e+05	2.451e+05	2.536e+05	1
161	956.50000	2.492e+05	2.496e+05	-411.7364	-0.165219	2.492e+05	2.5e+05	2.454e+05	2.539e+05	1
162	956.75000	2.495e+05	2.499e+05	-417.2852	-0.167246	2.495e+05	2.504e+05	2.457e+05	2.542e+05	1
163	957.00000	2.495e+05	2.502e+05	-767.8671	-0.307815	2.498e+05	2.507e+05	2.46e+05	2.545e+05	1
164	957.25000	2.491e+05	2.505e+05	-1379.459	-0.553668	2.501e+05	2.51e+05	2.463e+05	2.548e+05	1
165	957.50000	2.495e+05	2.508e+05	-1335.054	-0.535098	2.504e+05	2.513e+05	2.466e+05	2.551e+05	1
166	957.75000	2.5e+05	2.511e+05	-1117.649	-0.447027	2.507e+05	2.516e+05	2.469e+05	2.554e+05	1
167	958.00000	2.49e+05	2.514e+05	-2468.244	-0.991378	2.51e+05	2.519e+05	2.472e+05	2.557e+05	1
168	958.25000	2.492e+05	2.517e+05	-2578.840	-1.034997	2.513e+05	2.522e+05	2.475e+05	2.56e+05	1
169	958.50000	2.502e+05	2.52e+05	-1872.436	-0.748453	2.516e+05	2.525e+05	2.478e+05	2.563e+05	1
170	958.75000	2.499e+05	2.524e+05	-2401.031	-0.960608	2.519e+05	2.528e+05	2.481e+05	2.566e+05	1
171	959.00000	2.499e+05	2.527e+05	-2705.627	-1.082476	2.522e+05	2.531e+05	2.484e+05	2.569e+05	1
172	959.25000	2.502e+05	2.53e+05	-2796.222	-1.117769	2.525e+05	2.534e+05	2.487e+05	2.572e+05	1
173	959.50000	2.498e+05	2.533e+05	-3428.818	-1.372449	2.528e+05	2.537e+05	2.49e+05	2.575e+05	1
174	959.75000	2.499e+05	2.536e+05	-3657.414	-1.463510	2.531e+05	2.541e+05	2.493e+05	2.578e+05	1
175	960.00000	2.506e+05	2.539e+05	-3282.009	-1.309734	2.534e+05	2.544e+05	2.496e+05	2.581e+05	1
176	960.25000	2.502e+05	2.542e+05	-3943.605	-1.576005	2.537e+05	2.547e+05	2.499e+05	2.584e+05	1
177	960.50000	2.505e+05	2.545e+05	-3960.200	-1.580824	2.54e+05	2.55e+05	2.502e+05	2.587e+05	1
178	960.75000	2.516e+05	2.548e+05	-3132.796	-1.244922	2.543e+05	2.553e+05	2.505e+05	2.59e+05	1
179	961.00000	2.523e+05	2.551e+05	-2764.391	-1.095598	2.546e+05	2.556e+05	2.508e+05	2.593e+05	1
180	961.25000	2.525e+05	2.554e+05	-2900.987	-1.148974	2.549e+05	2.559e+05	2.511e+05	2.596e+05	1
181	961.50000	2.53e+05	2.557e+05	-2718.583	-1.074662	2.552e+05	2.562e+05	2.514e+05	2.599e+05	1
182	961.75000	2.536e+05	2.56e+05	-2413.178	-0.951644	2.555e+05	2.565e+05	2.517e+05	2.602e+05	1
183	962.00000	2.548e+05	2.563e+05	-1449.774	-0.568880	2.558e+05	2.568e+05	2.52e+05	2.606e+05	1
184	962.25000	2.545e+05	2.566e+05	-2137.369	-0.839953	2.561e+05	2.571e+05	2.523e+05	2.609e+05	1
185	962.50000	2.549e+05	2.569e+05	-1957.965	-0.767992	2.563e+05	2.575e+05	2.526e+05	2.612e+05	1
186	962.75000	2.553e+05	2.572e+05	-1886.561	-0.738898	2.566e+05	2.578e+05	2.53e+05	2.615e+05	1
187	963.00000	2.56e+05	2.575e+05	-1533.156	-0.598941	2.569e+05	2.581e+05	2.533e+05	2.618e+05	1
188	963.25000	2.569e+05	2.578e+05	-930.7518	-0.362324	2.572e+05	2.584e+05	2.536e+05	2.621e+05	1
189	963.50000	2.574e+05	2.581e+05	-672.3474	-0.261161	2.575e+05	2.587e+05	2.539e+05	2.624e+05	1
190	963.75000	2.577e+05	2.584e+05	-726.9430	-0.282094	2.578e+05	2.59e+05	2.542e+05	2.627e+05	1
191	964.00000	2.583e+05	2.587e+05	-376.5387	-0.145748	2.581e+05	2.593e+05	2.545e+05	2.63e+05	1
192	964.25000	2.59e+05	2.59e+05	-29.13465	-0.011249	2.584e+05	2.596e+05	2.548e+05	2.633e+05	1
193	964.50000	2.593e+05	2.593e+05	-66.73167	-0.025739	2.587e+05	2.599e+05	2.551e+05	2.636e+05	1

194	964.75000	2.6e+05	2.596e+05	388.66763	0.1494732	2.59e+05	2.603e+05	2.554e+05	2.639e+05	1
195	965.00000	2.604e+05	2.599e+05	451.05475	0.1732221	2.593e+05	2.606e+05	2.557e+05	2.642e+05	1
196	965.25000	2.609e+05	2.602e+05	611.40402	0.2343846	2.596e+05	2.609e+05	2.56e+05	2.645e+05	1
197	965.50000	2.608e+05	2.605e+05	282.64264	0.1083628	2.599e+05	2.612e+05	2.563e+05	2.648e+05	1
198	965.75000	2.617e+05	2.609e+05	844.57729	0.3227322	2.602e+05	2.615e+05	2.566e+05	2.651e+05	1
199	966.00000	2.619e+05	2.612e+05	746.72766	0.2851161	2.605e+05	2.618e+05	2.569e+05	2.654e+05	1
200	966.25000	2.634e+05	2.615e+05	1913.9791	0.7267070	2.608e+05	2.621e+05	2.572e+05	2.657e+05	1
201	966.50000	2.633e+05	2.618e+05	1476.9200	0.5610311	2.611e+05	2.624e+05	2.575e+05	2.66e+05	1
202	966.75000	2.647e+05	2.621e+05	2588.6992	0.9780376	2.614e+05	2.628e+05	2.578e+05	2.664e+05	1
203	967.00000	2.653e+05	2.624e+05	2834.2742	1.0684609	2.618e+05	2.631e+05	2.582e+05	2.667e+05	1
204	967.25000	2.653e+05	2.628e+05	2496.1005	0.9408560	2.621e+05	2.635e+05	2.585e+05	2.671e+05	1
205	967.50000	2.658e+05	2.632e+05	2566.7084	0.9656467	2.626e+05	2.639e+05	2.59e+05	2.675e+05	1
206	967.75000	2.676e+05	2.638e+05	3880.2062	1.4497910	2.631e+05	2.645e+05	2.595e+05	2.68e+05	1
207	968.00000	2.678e+05	2.644e+05	3416.3676	1.2755446	2.637e+05	2.652e+05	2.601e+05	2.687e+05	1
208	968.25000	2.684e+05	2.653e+05	3103.1831	1.1563121	2.644e+05	2.661e+05	2.61e+05	2.696e+05	1
209	968.50000	2.695e+05	2.663e+05	3144.0770	1.1667113	2.653e+05	2.674e+05	2.62e+05	2.707e+05	1
210	968.75000	2.697e+05	2.677e+05	2023.0651	0.7501836	2.664e+05	2.689e+05	2.632e+05	2.721e+05	1
211	969.00000	2.697e+05	2.692e+05	529.24671	0.1962237	2.677e+05	2.707e+05	2.647e+05	2.737e+05	1
212	969.25000	2.717e+05	2.709e+05	823.30980	0.3030384	2.692e+05	2.725e+05	2.663e+05	2.754e+05	1
213	969.50000	2.717e+05	2.725e+05	-875.1037	-0.322119	2.708e+05	2.743e+05	2.68e+05	2.771e+05	1
214	969.75000	2.727e+05	2.741e+05	-1337.885	-0.490547	2.724e+05	2.758e+05	2.695e+05	2.786e+05	1
215	970.00000	2.739e+05	2.753e+05	-1394.818	-0.509305	2.736e+05	2.769e+05	2.707e+05	2.798e+05	1
216	970.25000	2.736e+05	2.76e+05	-2383.917	-0.871327	2.744e+05	2.776e+05	2.715e+05	2.805e+05	1
217	970.50000	2.744e+05	2.762e+05	-1761.023	-0.641782	2.745e+05	2.778e+05	2.716e+05	2.807e+05	1
218	970.75000	2.753e+05	2.758e+05	-518.1448	-0.188214	2.74e+05	2.776e+05	2.712e+05	2.804e+05	1
219	971.00000	2.767e+05	2.751e+05	1604.2523	0.5798642	2.732e+05	2.77e+05	2.704e+05	2.797e+05	1
220	971.25000	2.762e+05	2.74e+05	2202.8655	0.7974318	2.721e+05	2.76e+05	2.694e+05	2.787e+05	1
221	971.50000	2.763e+05	2.729e+05	3396.0497	1.2289122	2.711e+05	2.748e+05	2.684e+05	2.775e+05	1
